# AdvFaceGAN: a face dual-identity impersonation attack method based on generative adversarial networks

**DOI:** 10.7717/peerj-cs.2904

**Published:** 2025-06-11

**Authors:** Hong Huang, Yang Yang, Yunfei Wang

**Affiliations:** School of Computer Science and Engineering, Sichuan University of Science & Engineering, Yibin, Sichuan, China

**Keywords:** Adversarial examples, Generative adversarial network, Face recognition model, Face matching robustness

## Abstract

This article aims to reveal security vulnerabilities in current commercial facial recognition systems and promote advancements in facial recognition technology security. Previous research on both digital-domain and physical-domain attacks has lacked consideration of real-world attack scenarios: Digital-domain attacks with good stealthiness often fail to achieve physical implementation, while wearable-based physical-domain attacks typically appear unnatural and cannot evade human visual inspection. We propose AdvFaceGAN, a generative adversarial network (GAN)-based impersonation attack method that generates dual-identity adversarial faces capable of bypassing defenses and being uploaded to facial recognition system databases in our proposed attack scenario, thereby achieving dual-identity impersonation attacks. To enhance visual quality, AdvFaceGAN introduces a structural similarity loss in addition to conventional generative loss and perturbation loss, optimizing the generation pattern of adversarial perturbations. Under the combined effect of these three losses, our method produces adversarial faces with excellent stealthiness that can pass administrator’s human review. To improve attack effectiveness, AdvFaceGAN employs an ensemble of facial recognition models with maximum model diversity to calculate identity loss, thereby enhancing similarity to target identities. Innovatively, we incorporate source identity loss into the identity loss calculation, discovering that minor reductions in target identity similarity can be traded for significant improvements in source identity similarity, thus making the adversarial faces generated by our method highly similar to both the source identity and the target identity, addressing limitations in existing impersonation attack methods. Experimental results demonstrate that in black-box attack scenarios, AdvFaceGAN-generated adversarial faces exhibit better stealthiness and stronger transferability compared to existing methods, achieving superior traditional and dual-identity impersonation attack success rates across multiple black-box facial recognition models and three commercial facial recognition application programming interfaces (APIs).

## Introduction

From unlocking smartphones to airport security, the widespread adoption of facial recognition systems is evident. In recent years, with the development and extensive application of deep neural networks (DNNs) (Hangaragi, Singh & N, 2023; Kortli et al., 2020; Rai et al., 2023), a considerable number of facial recognition models have been proposed and implemented (Alansari et al., 2023; Hangaragi, Singh & N, 2023; Liu et al., 2023). However, it is also crucial to note that research has demonstrated vulnerabilities in deep learning models used for facial recognition, such as susceptibility to adversarial attacks and backdoor attacks (Gu et al., 2023; Huang et al., 2024). The forged faces pose a significant challenge to security systems and privacy protection (Sharma, Kumar & Sharma, 2024). These vulnerabilities are similarly present in deep learning models for facial recognition tasks. By generating facial adversarial examples, attackers can achieve evasion attacks (where attackers remain undetected), obfuscation attacks (misclassifying attackers as other individuals), and impersonation attacks (misclassifying attackers as specified individuals) (Wang et al., 2023). Therefore, studying the characteristics and generation methods of facial adversarial examples is crucial for enhancing the robustness of facial recognition models against real-world attacks and threats.

Existing research on adversarial attacks using facial adversarial examples typically relies on three generation methods: gradient-based methods, optimization-based methods, and generative adversarial network (GAN)-based methods. Gradient-based methods generate perturbations based on the gradient direction of the model’s loss function, offering fast generation speeds and high effectiveness but are limited by the feasibility of obtaining the target model’s gradients. Moreover, attacks transferred using alternate model gradients exhibit weak transferability, leading to reduced attack effectiveness and insufficient visual realism, making them easily detectable by the human eye. Optimization-based methods generate adversarial examples with good visual realism and perform well in terms of attack effectiveness but suffer from slow optimization processes and high computational costs. GAN-based methods involve adversarial training between a Generator, Discriminator, and facial substitute model to learn the probability distribution of facial images. These methods offer fast generation speeds, high visual realism, and strong transferability of adversarial examples. However, they are prone to gradient vanishing or exploding during training, leading to failure.

In recent studies, Baluja & Fischer (2018) proposed the ATN network for adversarial example generation in image classification model, summarizing two modes: Perturbation-ATN (P-ATN) and adversarial autoencoding (AAE). The P-ATN mode advocates training residual modules to generate additive perturbations superimposed onto the source face. This method preserves much of the source face’s information, making the perturbations imperceptible to the naked eye. Subsequently, in the field of adversarial example generation for image classification, the representative work of the P-ATN mode is AdvGAN (Xiao et al., 2018). AdvGAN established the basic architecture of the Generator, Discriminator, and substitute model, and introduced a hyperparameter constant *ɛ* in the perturbation loss to effectively control the magnitude of the perturbation by adjusting c. In the field of adversarial face generation, a representative work of the P-ATN mode is AdvFaces (Deb, Zhang & Jain, 2020). AdvFace also generates global additive perturbations and evaluates the attack effectiveness on offline facial recognition models in black-box scenarios. However, it uses only Facenet as the auxiliary discriminator, which significantly limits the effectiveness of the perturbation attack. M3D (Zhao et al., 2023) discovering that minimizing the maximum model discrepancy between two substitute models could generate highly transferable adversarial examples.

Building on previous research, this article proposes a GAN-based impersonation attack method, AdvFaceGAN. Compared to prior work, our method targets impersonation attacks by integrating multiple face recognition models with maximum model discrepancy as auxiliary discriminators during training. This enhances both the aggressiveness and transferability of the generated adversarial faces and incorporates a structural similarity loss to guide the Generator in learning stealthier perturbation patterns, reducing the visual difference between the adversarial face and the source face. Finally, considering the practical attack scenarios in the threat model discussed later, the adversarial face needs to maintain sufficient feature similarity with the source face. Unlike previous methods, which overlook this aspect, our approach addresses it by adding a source identity loss to the common identity loss, thereby generating a “dual-identity” adversarial face with high feature similarity to both the source and target faces. The key contributions of this research are as follows:

1. A new impersonation attack method, AdvFaceGAN, is proposed to generate visually realistic adversarial faces, and own “dual identities” similar to both the source and target identities, achieves better transferability and success rate of traditional impersonation attacks than previous methods.

2. Propose a new success rate evaluation metric for dual-identity impersonation attacks that more accurately reflects real-world attack scenarios, and evaluate the effectiveness of previous methods using this metric. Additionally, simulate a dual-identity impersonation attack on a face recognition system in the physical domain, comparing the performance of previous methods and AdvFaceGAN, evaluated across three commercial face recognition APIs (Tencent, Aliyun, and Face++).

3. Open-sourcing AdvFaceGAN to GitHub serves as a reference for the subsequent research on the method of face adversarial sample generation based on generative adversarial networks, which promotes the development of the technology in this research direction.

## Related Works

### Generator adversarial network

Goodfellow et al. (2014) proposed generative adversarial networks, constructing fully-connected generator and discriminator networks. Through alternating training of Generator G and Discriminator D, the framework ultimately enables the Generator G, taking noise *z* as input, to produce samples matching the data distribution. However, fundamental issues in original GANs remained unresolved: training instability, inadequate guidance from loss metrics for training progression, and lack of diversity in generated samples.

Subsequent research efforts addressed these challenges. DCGAN (Radford et al. 2016) implementing convolutional architectures for both generator and discriminator. The convolutional layers effectively capture spatial structures and local features in images, significantly improving performance in image generation tasks. Wasserstein generative adversarial networks (WGAN) (Arjovsky & Bottou, 2017) theoretically demonstrating that the instability of traditional GANs stems from the Jensen–Shannon (JS) divergence becoming ineffective when the supports of distributions are disjoint or minimally overlapping, leading to gradient explosion or vanishing. Their solution employed Wasserstein distance as an alternative metric, which measures the minimum “transport cost” between distributions and provides smooth gradients even with disjoint supports. The practical implementation involved enforcing 1-Lipschitz constraints on the discriminator through weight clipping, leveraging Kantorovich-Rubinstein duality to transform Wasserstein distance optimization into a dual problem. WGAN’s modifications included: removing the final Sigmoid layer in the discriminator, eliminating logarithmic computations in loss functions, applying hard weight clipping with constant C, and recommending RMSProp over momentum-based optimizers. These changes substantially improved training stability, mitigated mode collapse, and ensured sample diversity. Nevertheless, weight clipping introduced two limitations: restricted network capacity due to parameter constraints, and potential gradient explosion/vanishing issues.

This led to the development of WGAN-GP (Gulrajani, Ishaan et al. 2017), which introduced a gradient penalty term in the discriminator loss. This penalty enforces soft constraints on the gradient norm of discriminator outputs for both real and generated samples, maintaining smooth gradients without explicit weight clipping. The gradient penalty approach preserved 1-Lipschitz continuity while avoiding clipping-induced performance degradation, enabling higher-quality image generation and compatibility with momentum-based optimizers like Adam for faster convergence.

### Adversarial attacks

This section reviews the development of adversarial attack techniques and related research on adversarial attacks for face recognition tasks. The methods for generating adversarial samples were first proposed in the field of image classification models, primarily including optimization-based methods, gradient-based methods, and generative adversarial network-based methods:

• **Optimization-based methods**: For example, C&W (Carlini & Wagner, 2017) improved the target optimization function in iterative attacks and gradually constrained the function within the disturbance magnitude limits, effectively reducing the perturbation size of adversarial samples while maintaining excellent visual quality.
• **Gradient-based methods**: For example, FGSM (Goodfellow, Shlens & Szegedy, 2014) used first-order gradients to efficiently compute adversarial perturbations. Although fast, this method tends to be unstable in its attack. Later methods such as PGD (Madry et al., 2017), BIM (Kurakin, Goodfellow & Bengio, 2018), and MI-FGSM (Dong et al., 2018) introduced iterative optimization steps, which enhanced the attack effectiveness of adversarial samples.
• **GAN-based methods**: For instance, Baluja & Fischer (2018) proposed the ATN network, summarizing two patterns for generating adversarial samples: P-ATN and AAE. The P-ATN pattern advocates training a residual module to generate additive perturbations with p-norm constraints over the source face. This preserves the overall information of the source face, but the perturbations are more apparent on the edges or corners of the image. The method in this paper is a variation of the P-ATN pattern. AdvGAN (Xiao et al., 2018), a representative work of the P-ATN pattern, determined the basic architecture for the Generator, Discriminator, and surrogate models, and introduced a hyperparameter *ɛ* into the perturbation loss, which can control the perturbation magnitude by adjusting *ɛ*.

The generation of adversarial examples for face recognition models includes traditional attack methods such as Fast Gradient Sign Method (FSGM), Momentum Iteration—Fast Gradient Sign Method (MI-FGSM), and Carlini & Wagner (C&W), which adapt surrogate models from image classification models to face recognition models to generate adversarial faces. Or newly developed attack methods for face recognition models, which can be further categorized into two approaches: local perturbation methods, which are more suited for physical-domai, and global perturbation methods, which are more applicable in the digital-domain.

• **Local perturbation-based methods**: AdvGlass (Sharif et al., 2016) applied an iterative gradient descent algorithm to generate adversarial glasses and incorporated smoothing and printer color gamut losses to make the adversarial glasses printable for physical-world attacks; AdvHat (Komkov & Petiushko, 2021) used the MI-FGSM method to generate adversarial hats; Adv-MakeUP (Yin et al., 2021) used GANs to generate adversarial makeup with good transferability but limited attack effectiveness; and in 2023, Yang et al. (2023) at the Conference on Computer Vision and Pattern Recognition (CVPR) conference proposed the design of adversarial textured 3D masks shortened as Adversarial Textured 3D meshes (AT3D) with complex topological structures, which were 3D printed and placed on an attacker’s face to evade defenses. The perturbations were generated using BIM and updated in the low-dimensional coefficient space of 3DMM. AT3D has the best attack effectiveness among local perturbation methods, but its concealment is still weak, as the mask has clearly visible edges. Both AT3D and Adv-MakeUP will be included as representative local perturbation methods for comparison in subsequent sections.
• **Global perturbation-based methods**: AdvFaces (Deb, Zhang & Jain, 2020) employed FaceNet as a surrogate model under the P-ATN pattern to generate high-quality adversarial faces for obfuscation or impersonation attacks. TIP-IM (Yang et al., 2021) enhanced the naturalness of adversarial faces by introducing a maximum mean discrepancy (MMD) loss based on MI-FGSM, and optimized attack efficacy through a greedy insertion algorithm targeting multiple facial identities. AMT-GAN (Hu et al., 2022) addressed the conflict between adversarial noise and cycle-consistency loss *via* a regularization module and joint training strategy, achieving notable performance improvements in black-box scenarios. Sibling-Attack (Li et al., 2023) leverages gradient information from facial attribute recognition tasks and integrates PGD-based perturbation generation to substantially boost attack success rates and transferability. DiffAM (Sun et al., 2024), as an enhancement to the Adversarial Makeup Transfer-GAN (AMT-GAN) framework, synergized the high-quality generation capability of diffusion models with CLIP’s fine-grained semantic control, demonstrating superior black-box attack success rates and naturalness compared to existing makeup transfer methods. AdvFaces, TIP-IM, Sibling-Attack, and DiffAM will be included as representative global perturbation methods in our comparative experiments.

### Threat model

**Attacker:** The insider employee uses open-source pre-trained face recognition models, open-source datasets, *etc.*, to build adversarial face generation technology. The insider employee is unaware of the model structure and parameters of the face recognition system or the datasets used for training. However, the insider employee has an image of the attacker’s face.

**Attack scenario:** Some face recognition systems, such as those used in attendance machines, allow administrators to upload face images of employees for registration. Administrators can request employees to submit their face images online and upload them to register or update personnel information.

**Attack goal:** The attacker is identified as the insider employee and is able to pass through the face recognition system without disrupting the insider employee’s normal authentication.

**Limitations:** The adversarial face must be uploaded to the face database.

**Figure 1:**
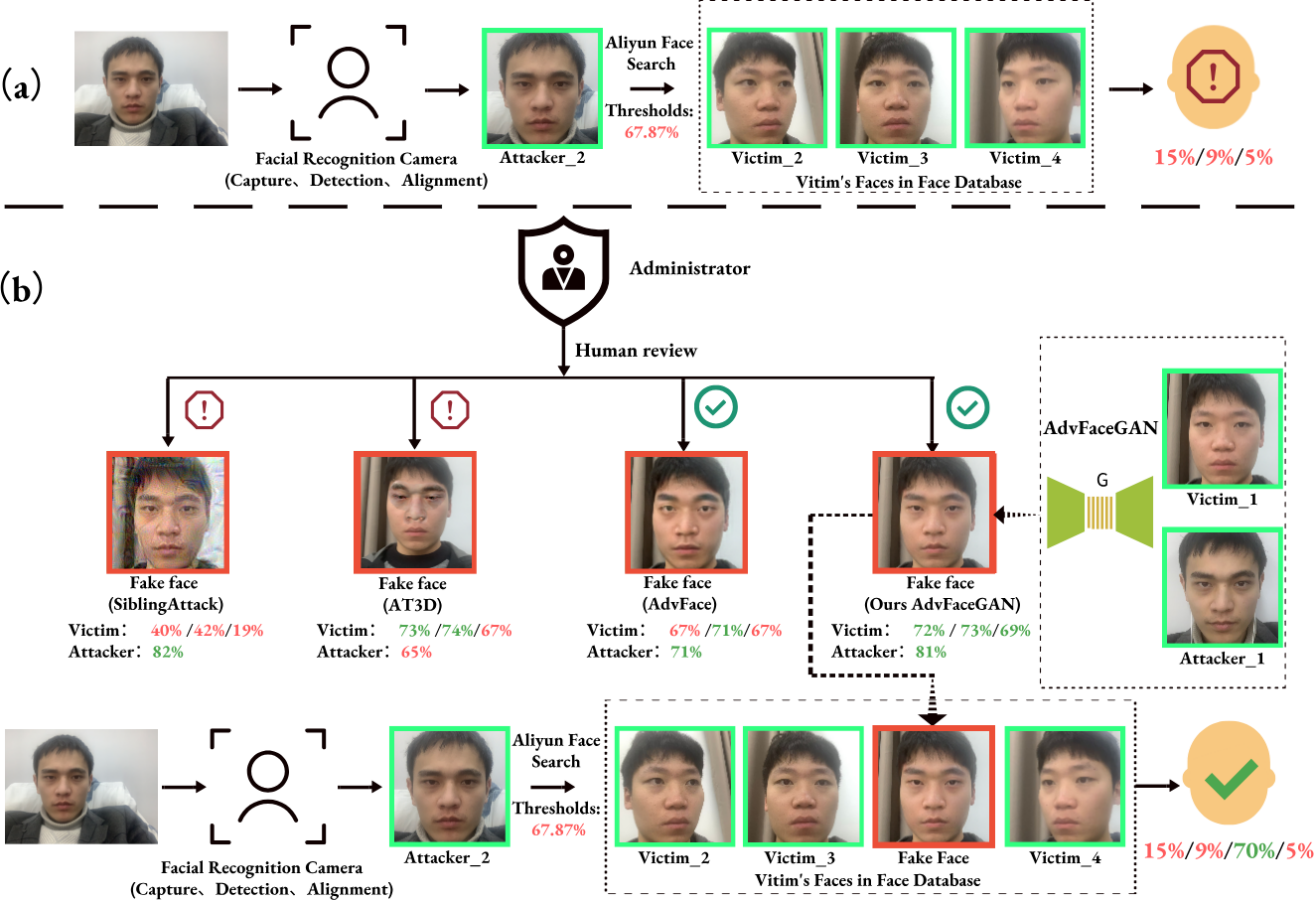
Threat model (A) normal matching process, (B) dual-identity impersonation attack process. The clean face’s border is in green, the adversarial face’s border is in red, the green number indicates that Aliyun’s Face API judges the feature similarity to be higher than the threshold, and the red is lower than the threshold.

**Attack process:** As shown in Fig. 1A, when the attacker’s face, Attacker_2, is compared to the three clean faces of the insider employee in the face database (Victim_2, Victim_3, and Victim_4), the feature similarity is all below 15% (as returned by the Aliyun Face Search API), which is far below the default threshold of 67.87% for Aliyun Face Search API, so direct matching with the insider employee fails. However, in Fig. 1B, the insider employee uses the AdvFaceGAN method in this paper to generate a “dual-identity” adversarial face, FakeFace, based on the attacker’s face, Attacker_1, and one of the insider employee’s faces, Victim_1. This adversarial face is submitted to the Administrator. Due to the high visual quality of FakeFace, it can pass the Administrator’s human review, and also pass defense against the upload when compared to the source face quality. Additionally, the feature similarity between FakeFace and the three existing faces of the insider employee in the database is 72%, 73%, and 69% (as returned by the Aliyun Face Compare API), which surpasses the 69% within-class similarity threshold recommended by the Aliyun AddFace API. As a result, FakeFace is successfully uploaded to the face database under the insider employee. When the attacker attempts to attack face recognition again, Attacker’s face Attacker_2 will have a 70% feature similarity to FakeFace, which is associated with the insider employee. Therefore, the attacker will be recognized as the insider employee and pass through the face recognition system, Meanwhile, during this period, the insider employees are able to pass the face recognition system normally.

It can be observed that under this threat model, the various liveness detection defenses set up during the face image capture in the face recognition system are cleverly bypassed. In contrast, some similar advanced attack methods either fail due to being too obvious to pass the Administrator’s visual inspection (*e.g.*, SiblingAttack, AT3D), or they cannot be successfully uploaded to the face database under the insider employee’s profile due to insufficient similarity to the source identity (*e.g.*, SiblingAttack, AdvFaces), or they fail to match the adversarial face with the target identity due to insufficient similarity (*e.g.*, AT3D), even if the upload is successful. Furthermore, if we assume the database Administrator is an insider or there is some vulnerability that grants the Attacker direct permission to upload faces to the face database, any employee in the database—even the CEO—could unknowingly and imperceptibly be impersonated by the attacker as an “insider.”

## Method

### AdvFaceGAN structure

**Figure 2:**
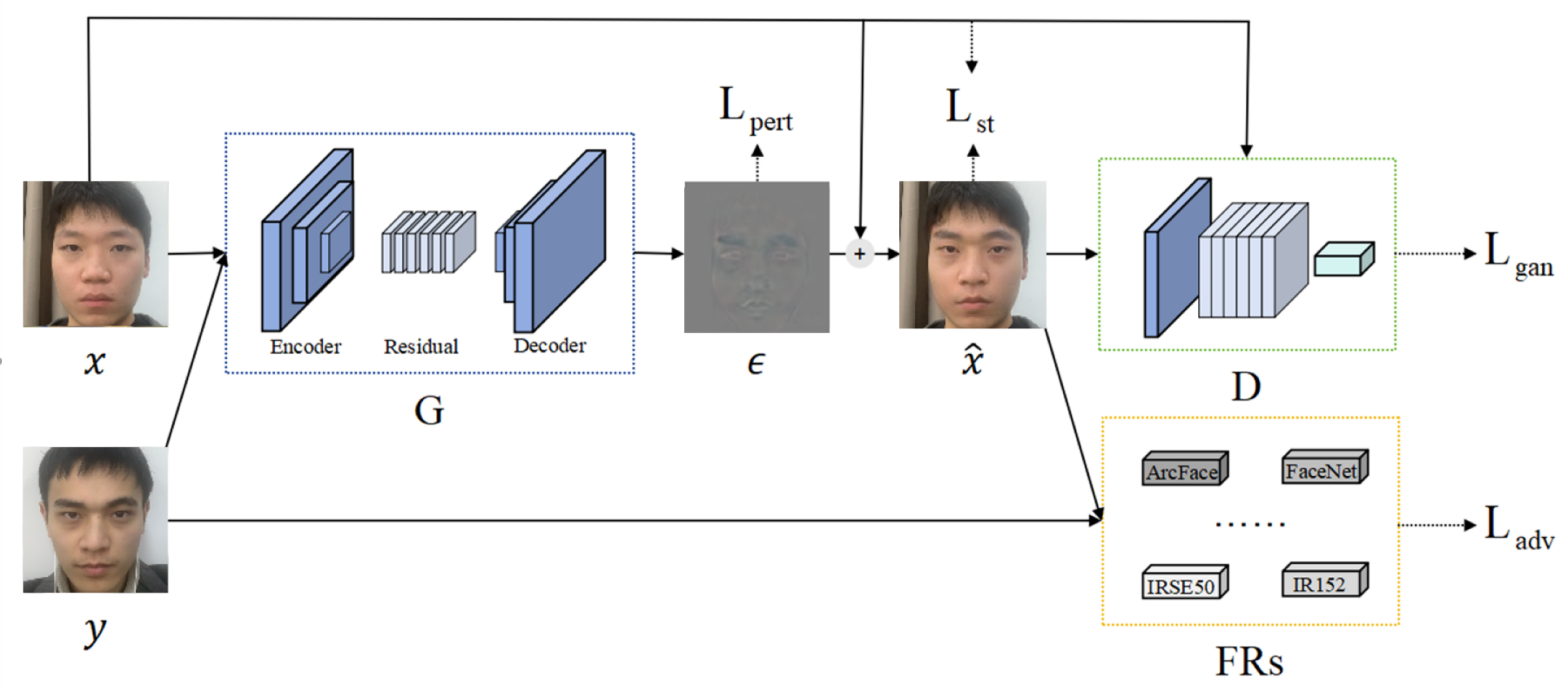
Structural diagram of AdvFaceGAN.

The overall structure of AdvFaceGAN, as shown in Fig. 2, consists of three components: a Discriminator *D*, a Generator *G*, and auxiliary discriminators *FRs*. The interplay between these components allows the adversarial perturbation *ϵ* generated by *G* to be added to the attacker’s face *x*, resulting in the adversarial face $\tilde {x}$, which remains in the same distribution as the real face *x*. Additionally, $\tilde {x}$ has enough similarity to both the attacker’s face *x* and the victim’s face *y*.

**Figure 3:**
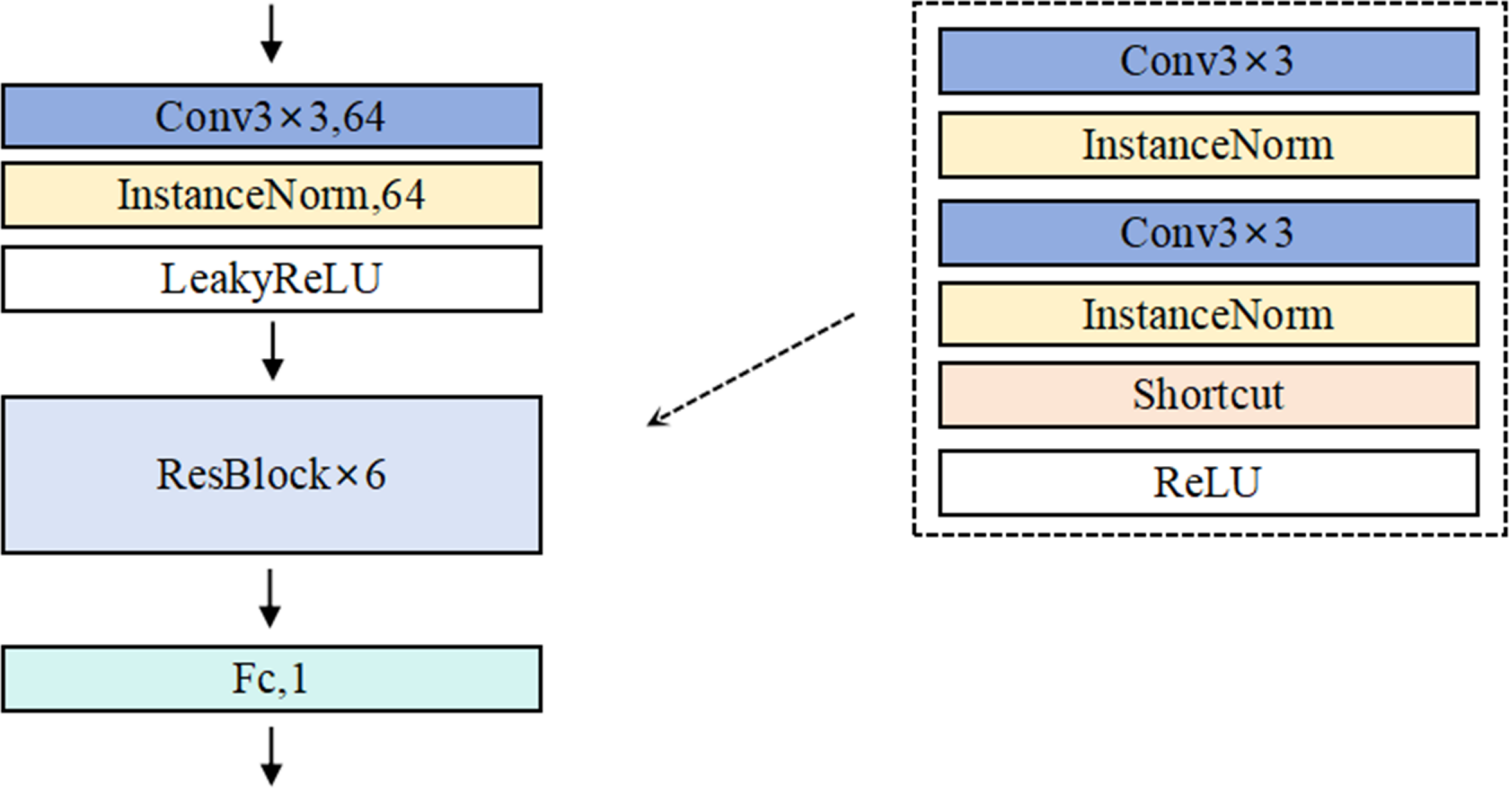
Schematic diagram of the Generator network structure.

The detailed architecture of Discriminator *D* is illustrated in Fig. 3. In the construction of the discriminator, AdvFaceGAN employs multiple residual convolutional layers for feature extraction and utilizes a final full connected layer to map the extracted features into a scalar output representing image authenticity. However, the introduction of gradient penalty imposes a critical architectural constraint: conventional batch normalization (BN) layers—commonly used in standard GANs and WGANs—are incompatible with this framework.

This incompatibility arises because gradient penalty requires a strictly sample-wise input–output relationship for accurate gradient calculation, whereas BN normalizes statistics across batch dimensions (*i.e.,* batch inputs → batch outputs). Such batch-level operations disrupt the per-sample gradient computation essential for effective gradient penalty enforcement. To resolve this conflict while maintaining normalization benefits, our method replaces BN with instance normalization (IN) in the discriminator.

**Figure 4:**
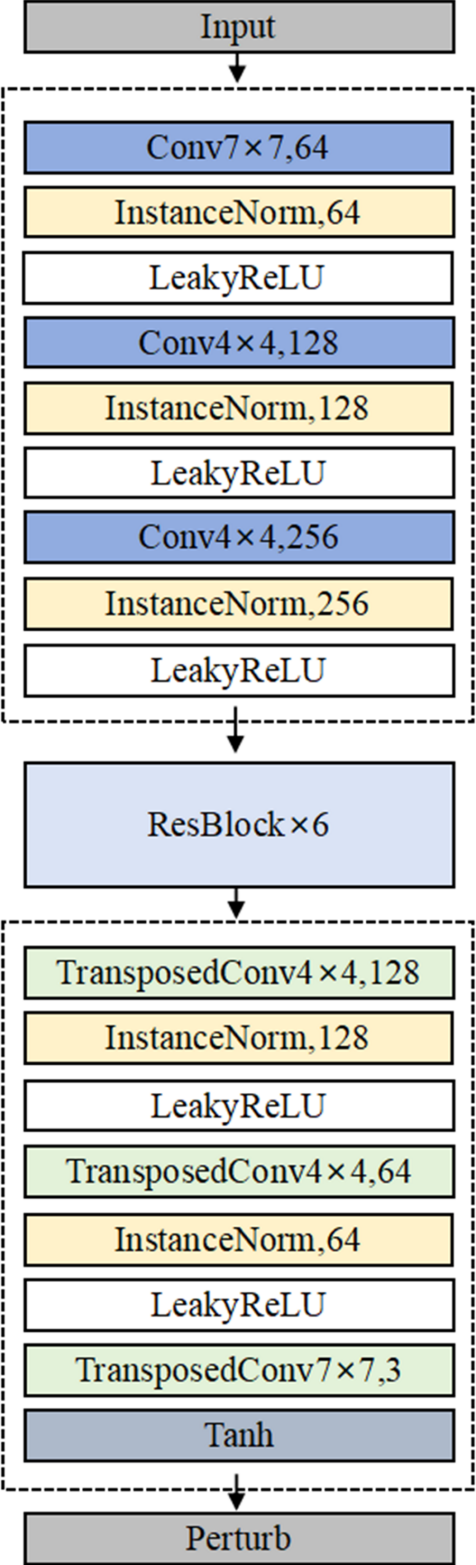
Integrated models achieve smaller generalization errors.

The detailed architecture of Generator G is illustrated in Fig. 4. In the generator architecture, AdvFaceGAN constructs an autoencoder integrated with residual blocks to synthesize adversarial perturbations *ϵ*. When processing an input pair comprising a source facial image *x* and a target facial image *y*, the Generator first concatenates *x* and *y* channel-wise. This concatenated input is then fed into an encoder module that progressively downscales the feature maps through multiple strided convolutional layers. The compressed latent representation subsequently passes through a series of residual blocks to learn deep-level discriminative features. Following this, a decoder module employing transposed convolutional layers gradually upscales the features to the original spatial dimensions. Finally, the output undergoes normalization *via* Tanh activation layer, producing an adversarial perturbation *ϵ* with identical dimensions to the input facial images.

### Ensemble of face recognition models

**Figure 5:**
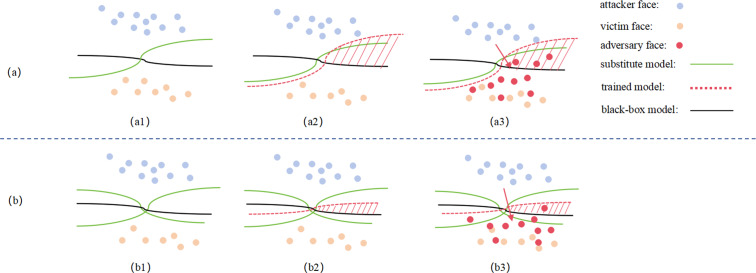
Schematic diagram of the Discriminator network structure.

In previous research, researchers typically computed the loss by using a single target model or by averaging multiple models. In our approach, inspired by the findings of M3D (Zhao et al., 2023), we reconsider the role of surrogate models in the generation of adversarial face samples. We introduce multiple face recognition surrogate models with maximal model differences to construct an ensemble white-box model, FRs. FRs acts as an auxiliary discriminator in the adversarial training process between the Generator and the Discriminator, enhancing the Generator’s ability to produce adversarial samples with a better generalization error bound. The principle is illustrated in Fig. 5.

As seen in Fig. 5A, when using a single surrogate model, the abstract decision boundary of the trained adversarial face (red dashed line) and the generalization error to the black-box model (red shaded area in a3) are significantly larger due to the surrogate and black-box model differences. This results in a notable loss in attack success rates. However, in Fig. 5B, when using multiple ensemble white-box models, the abstract decision boundary of the trained adversarial face becomes more aligned with the black-box model, reducing the generalization error (red shaded area in b3), thereby improving the effectiveness of black-box attacks.

### Training process design

In order to ensure stable convergence during the training process, we follow the recommendations of WGAN-GP and design the model structure and training details as follows: (1) adopting the Adam optimizer to improve performance; (2) enforcing a gradient penalty term with coefficient *λ* =10 in the discriminator’s loss function (Eq. (1)), ensuring 1-Lipschitz continuity between real and generated samples; (3) eliminating log-transformations in the loss calculations; (4) replacing BatchNorm layers with InstanceNorm in both the Generator *G* and Discriminator *D* architectures to ensure the effectiveness of the gradient penalty.

In order for the Discriminator *D* to learn to distinguish between adversarial faces and real faces, the loss function *L*_*D*_ is calculated as shown in Eq. (1):

(1) \begin{eqnarray*}{L}_{D}=E_{x\sim {P}_{g}} \left[ D \left( x \right) \right] -E_{x\sim {P}_{r}} \left[ D \left( x \right) \right] +\lambda E_{\hat {x}\sim \chi } \left[ { \left( { \left\| \nabla \hat {x}D \left( \hat {x} \right) \right\| }_{2}-1 \right) }^{2} \right] \end{eqnarray*}

(2) \begin{eqnarray*}\hat {x}=\alpha {x}_{r}+ \left( 1-\alpha \right) {x}_{g} \left( \alpha \sim U \left[ 0,1 \right] ,{x}_{r}\sim {P}_{r},{x}_{g}\sim {P}_{g} \right) \end{eqnarray*}

where *P*_*g*_ represents the space of adversarial samples, *P*_*r*_ represents the space of real samples, and $D \left( x \right) $ is the prediction value of the discriminator *D* for the face *x*. The final part is the gradient penalty term, where *λ* is a hyperparameter controlling the strength of the penalty, set to 10. *χ* is the overall sample space containing both the adversarial sample space *P*_*g*_ and the real sample space *P*_*r*_. The collection method for the intermediate sample $\hat {x}$ in the overall sample space is given by Eq. (2). Here, $\nabla \hat {x}D \left( \hat {x} \right) $ is the gradient of the discriminator for the intermediate sample $\hat {x}$.

For adversarial faces, we want $D \left( x \right) $ to be as small as possible, hence the positive sign in front of ${E}_{x\sim {P}_{g}} \left[ D \left( x \right) \right] $; for real faces, we want $D \left( x \right) $ to be as large as possible, hence the negative sign in front of ${E}_{x\sim {P}_{r}} \left[ D \left( x \right) \right] $. The numerical space of the discriminator’s gradient is the overall sample space, which is of high dimensionality and difficult to compute. Therefore, we sample real and adversarial intermediate samples $\hat {x}$ from each batch to compute the penalty term: $E_{\hat {x}\sim \chi } \left[ { \left( { \left\| \nabla \hat {x}D \left( \hat {x} \right) \right\| }_{2}-1 \right) }^{2} \right] $. This ensures that the *L*_2_ norm of $D \left( \hat {x} \right) $ is as close to 1 as possible, maintaining the Lipschitz continuity of the discriminator function. Optimizing *L*_*D*_ allows the discriminator to learn to differentiate between adversarial faces and real faces.

In order for the Generator *G* to learn to generate adversarial face samples with high attack capability and high stealthiness, the Generator’s loss function is obtained by adding four types of losses. The convergence goal of the model is controlled by setting weight hyperparameters for the losses. *L*_*G*_’s calculation is shown in Eq. (3):

(3) \begin{eqnarray*}{L}_{G}={L}_{GAN}+{\lambda }_{pert}{L}_{Pert}+{\lambda }_{adv}{L}_{adv}+{\lambda }_{st}{L}_{st}.\end{eqnarray*}

In the design of the Generator’s loss function, we incorporate four types of losses to form the total loss. These include the adversarial loss *L*_*gan*_ from the discriminator *D*. By fixing the parameters of discriminator *D* and optimizing the Generator according to *L*_*gan*_, we ensure that the adversarial face $\tilde {x}=x+G(x)$ is closer to real face’s distribution. The calculation of *L*_*gan*_ is given by Eq. (4):

(4) \begin{eqnarray*}{L}_{gan}=-E_{x\sim {P}_{r}} \left( D \left( x+G \left( x \right) \right) \right) .\end{eqnarray*}

The perturbation loss *L*_*Pert*_ from the adversarial perturbation *ϵ* = *G*(*x*, *y*), doing the perturbation amount calculation with two-paradigm numbers can effectively penalize the perturbation at each pixel point so as to avoid useless perturbation, and control the perturbation amount by max to be unpunished at low perturbation upper limit *ɛ*, thus encouraging the learning of perturbation and accelerating the model convergence. The computation of *L*_*pert*_ is shown in Eq. (5):

(5) \begin{eqnarray*}{L}_{pert}=E_{x,y\sim {P}_{r}} \left[ \max _{} \left( ,{ \left\| \epsilon \right\| }_{2} \right) \right] =E_{x,y\sim {P}_{r}} \left[ \max _{} \left( ,{ \left\| G \left( x,y \right) \right\| }_{2} \right) \right] \end{eqnarray*}

where ${ \left\| G \left( x \right) \right\| }_{2}$ indicates the perturbation amount calculated from *ϵ* = *G*(*x*) using the ${ \left\| \cdot \right\| }_{2}$ norm, and *ɛ* denotes the upper limit on perturbation.

The structural loss *L*_*st*_ from the structural similarity between the source face *x* and the adversarial face $\tilde {x}=x+G(x,y)$ and is controlled by max so that the structural similarity is not penalized when it is higher than the lower limit of the structural similarity *ζ*. *L*_*st*_ is computed as in Eq. (6):

\begin{eqnarray*}{L}_{st}=E_{x\sim {P}_{r},\tilde {x}\sim {P}_{g}} \left[ \max _{} \left( 1-\zeta ,1-ssim \left( x,\tilde {x} \right) \right) \right] \end{eqnarray*}

(6) \begin{eqnarray*}=E_{x,y\sim {P}_{r}} \left[ \max _{} \left( 1-\zeta ,1-ssim \left( x,x+G(x,y) \right) \right) \right] \end{eqnarray*}

where *ssim*(⋅, ⋅) denotes the structural_similarity_index_measure function provided by torchmetrics and *ζ* denotes the lower limit of structural similarity.

The identity loss *L*_*adv*_ from the auxiliary discriminator FRS combines the source identity loss *L*_*adv*_*source*_ and the target identity loss *L*_*adv*_*target*_, statistically predicts the losses of multiple face recognition models with the largest differences, and by adjusting the source identity loss weights *η*, it can make the generated antagonistic faces have a balanced “dual identity”, *L*_*adv*_ is calculated as in Eq. (7):

\begin{eqnarray*}{L}_{adv}=\eta \ast {L}_{ad{v}_{source}}+{L}_{ad{v}_{target}} \end{eqnarray*}

    $=E_{x,y\sim {P}_{r},\tilde {x}\sim {P}_{g}} \left[ \eta \ast \frac{{\mathop{\sum }\nolimits }_{i=1}^{M} \left( 1-\cos \left[ {f}_{i} \left( \tilde {x} \right) ,{f}_{i} \left( x \right) \right] \right) }{M} + \frac{{\mathop{\sum }\nolimits }_{i=1}^{M} \left( 1-\cos \left[ {f}_{i} \left( \tilde {x} \right) ,{f}_{i} \left( y \right) \right] \right) }{M} \right] $

(7) \begin{eqnarray*} =E_{x,y\sim {P}_{r}} \left[ \eta \ast \frac{\sum _{i=1}^{M} \left( 1-\cos \nolimits \left[ {f}_{i} \left( x+G \left( x,y \right) \right) ,{f}_{i} \left( x \right) \right] \right) }{M} \right. \nonumber\\\displaystyle  + \left. \frac{\sum _{i=1}^{M} \left( 1-\cos \nolimits \left[ {f}_{i} \left( x+G \left( x,y \right) \right) ,{f}_{i} \left( y \right) \right] \right) }{M} \right] \end{eqnarray*}

where *x* denotes the source face, $\tilde {x}=x+G(x,y)$ denotes the adversarial face, *y* denotes the target face, M denotes the number of white-box substitution models integrated by FRS, *f*_*i*_(⋅) denotes the feature vector computation function corresponding to the ith fixed-parameter white-box substitution model, and *cos*(⋅, ⋅) denotes the cosine similarity computation function of two features.

The pseudocode for the training process of AdvFaceGAN is shown in Table 1.

**Table 1:** AdvFaceGAN’s training process.

Input:source face *x*, target face *y*. Output:The parameters of *θ*_*D*_ and *θ*_*G*_ after convergence.
1 Initialize parameters of *θ*_*D*_ and*θ*_*G*_,load Dataset
2 while *L*_*adv*_*t*arg*et*_ > *L*_*adv*_*source*_ do:
3 for c in epoch_size do:
4 Get batch with x,y from Dataset
5 *ϵ* = *G*(*x*, *y*)
6 $\widetilde {x}=x+\epsilon $
7 $\frown x=\sigma x+(1-\sigma )\widetilde {x}$
8 ${L}_{D}= \frac{1}{\mathrm{m}} [{\mathop{\sum }\nolimits }_{i=1}^{m}D({\widetilde {x}}_{i})]- \frac{1}{\mathrm{m}} [{\mathop{\sum }\nolimits }_{i=1}^{m}D({x}_{i})]+{\lambda }_{gp} \frac{1}{\mathrm{m}} [{\mathop{\sum }\nolimits }_{i=1}^{m}({ \left\| {\nabla }_{\frown {x}_{i}}D(\frown {x}_{i}) \right\| }_{2}-1)^{2}]$
9 *θ*_*D*_ = *Adam*(∇_*D*_*L*_*D*_, *θ*_*D*_, *β*_1_, *β*_2_, *lr*, *weight*_*decay*)
10 ${L}_{gan}=- \frac{1}{m} [{\mathop{\sum }\nolimits }_{i=1}^{m}D(\widetilde {x})]$
11 ${L}_{adv\text{_}source}= \frac{1}{T\ast m} {\mathop{\sum }\nolimits }_{i=1}^{m}{\mathop{\sum }\nolimits }_{j=1}^{T}(1-\cos [{f}_{j}({\widetilde {x}}_{i}),{f}_{j}({x}_{i})])$
12 ${L}_{adv\text{_}target}= \frac{1}{T\ast m} {\mathop{\sum }\nolimits }_{i=1}^{m}{\mathop{\sum }\nolimits }_{j=1}^{T}(1-\cos [{f}_{j}({\widetilde {x}}_{i}),{f}_{j}({y}_{i})])$
13 *L*_*adv*_ = *η*∗*L*_*adv*_*source*_ + *L*_*adv*_*target*_
14 ${L}_{pert}= \frac{1}{m} {\mathop{\sum }\nolimits }_{i=1}^{m}max(,{ \left\| {\epsilon }_{i} \right\| }_{2})$
15 ${L}_{st}= \frac{1}{m} {\mathop{\sum }\nolimits }_{i=1}^{m}\max (1-\zeta ,1-ssim({x}_{i},{\widetilde {x}}_{i}))$
16 *L*_*G*_ = *L*_*gan*_ + *λ*_*pert*_*L*_*pert*_ + *λ*_*adv*_*L*_*adv*_ + *λ*_*st*_*L*_*st*_
17 *θ*_*G*_ = *Adam*(∇_*G*_*L*_*G*_, *θ*_*G*_, *β*_1_, *β*_2_, *lr*, *weight*_*decay*)
18 end for
19 save *θ*_*D*_ and *θ*_*G*_
20 end while

## Experiments

### Evaluation metrics

In order to reflect the advantages of the adversarial faces generated by the method in this paper in terms of visual quality and attack effect, we design the following evaluation metrics:

Visual quality metrics: we take the structural similarity (SSIM) peak signal-to-noise ratio (PSNR), mean squared error (MSE) of the source face and the adversarial face as the visual quality evaluation metric. The range of structural similarity is [0, 1], when two images are the same, the value is 1. The larger the image structure gap, the lower the value, and it is generally considered to be greater than 0.92 to satisfy the visual similarity. The peak signal-to-noise ratio evaluates the image quality, the larger the better, greater than 30dB, the human eye is difficult to detect the difference between the compressed and the source face. MSE intuitively indicates the magnitude of the image change, the smaller the weaker the change. The specific formulas for the three indicators are shown in Eqs. (8), (9) and (10):

(8) \begin{eqnarray*}\text{SSIM} \left( x,y \right) = \frac{ \left( 2{\mu }_{x}{\mu }_{y}+{c}_{1} \right) \left( 2{\sigma }_{xy}+{c}_{2} \right) }{ \left( {\mu }_{x}^{2}+{\mu }_{y}^{2}+{c}_{1} \right) \left( {\sigma }_{x}^{2}+{\sigma }_{y}^{2}+{c}_{2} \right) } \end{eqnarray*}

(9) \begin{eqnarray*}PSNR=20{\log \nolimits }_{10} \left( \frac{MA{X}_{I}^{2}}{\sqrt{MSE}} \right) \end{eqnarray*}

(10) \begin{eqnarray*}MSE= \frac{1}{mn} \sum _{i=0}^{m-1}\cdot \sum _{j=0}^{n-1}[I \left( i,j \right) -K \left( i,j \right) ]^{2}\end{eqnarray*}

where, *x*, *y* are comparison images, *μ*_*x*_, *μ*_*y*_, ${\sigma }_{x}^{2}$, ${\sigma }_{y}^{2}$ are the mean and variance of x and y, ${\sigma }_{y}^{x}$ is the covariance of *x* and *y*, ${c}_{i}={ \left( {k}_{i}L \right) }^{2}(i=1,2)$ is the constant used to maintain the stability, where *k*_1_ = 0.01, *k*_2_ = 0.03, and *L* is the range of pixel values of the image. $I \left( i,j \right) $ is the adversarial face, *K*(*i*, *j*) is the source face, *MAX*_*I*_ is the maximum possible pixel value of the image and mn is the size of the image.

Attack effectiveness metrics: We use the attack success rate (ASR) of the attack on the face recognition model as the evaluation metrics of the attack effectiveness, and the ASR aims at calculating the ratio of the successful realization of the attack among *N* pairs *x*_*s*_ ∼ *x*_*t*_. In the traditional impersonation attack, the cosine similarity between the antagonist face features and the target non-homo face features is greater than the recognition threshold is considered as successful impersonation, which is calculated as shown in Eq. (11):

(11) \begin{eqnarray*}AS{R}_{1}= \frac{\sum _{i}^{N}{1}_{\tau } \left( \cos \nolimits \left[ f \left( {x}_{i}+G({x}_{i},{y}_{i}) \right) ,f \left( {y}_{i} \right) \right] > \tau \right) }{N} \times 100\%\end{eqnarray*}

where 1_*τ*_ is the indicator function, *x* is the source face, *y* is the target face, $G \left( \mathrm{ \ast } \right) $ is the pre-trained generator, *f*( ∗) is the target black-box face recognition model, *f*(*x*) is the output face features after inputting the face *x*, *cos*[*a*, *b*] denotes the computation of the cosine similarity between the two face features, and *τ* is the recognition threshold of *f* at 0.1% FAR or 0.01% FAR (False Accept Rate) under the recognition threshold. *N* is the total number of image pairs.

This article found that the face recognition system database often requires that the uploaded face and other faces in the person’s face database are higher than a certain threshold, so it further explores the success rate of the attack in the case where the antagonist face is required to maintain a high degree of similarity with the source identity, *i.e.,* the cosine similarity between the antagonist face features and the source same-person face features and the target nonhomo-person face features are both greater than the recognition threshold is considered to be successful in the impersonation, and its formula is shown in Eq. (12) shows:

(12) \begin{eqnarray*}AS{R}_{2}= \frac{\sum _{i}^{N}{1}_{\tau } \left( \cos \nolimits \left[ f \left( {x}_{i}+G \left( {x}_{i},{y}_{i} \right) \right) ,f \left( {x}_{i} \right) \right] > \tau \mathrm{{\XMLAMP}}\mathrm{{\XMLAMP}}\cos \nolimits \left[ f \left( {x}_{i}+G({x}_{i},{y}_{i}) \right) ,f \left( {y}_{i} \right) \right] > \tau \right) }{N} \nonumber\\\displaystyle  \times 100\%.\end{eqnarray*}

However, only using the above two attack success rates as the attack effect evaluation metric is still insufficient, because whether the attack is successful is based on whether the feature similarity is greater than the model’s recognition threshold, which is a discrete metric. Assuming that the recognition threshold of the face recognition model is 30%, an antagonistic sample with 70% similarity to the target face and an antagonistic sample with 50% similarity to the target face are equivalent in the statistics of *ASR*_1_, however, it is obvious that the former achieves a better attack effect. Therefore, when evaluating the attack effect, we add the average similarity of the output of the target face recognition model, which is a continuous metric that can compare the attack effect more effectively. The average similarity with the source identity (Fake & Source Similarity, FSS) and the average similarity with the target identity (Fake & Target Similarity, FTS) under the face recognition model f are shown in Eqs. (13) and (14).

(13) \begin{eqnarray*}FSS= \frac{\sum _{i}^{N}\cos \nolimits \left[ f \left( {x}_{i}+G \left( {x}_{i},{y}_{i} \right) \right) ,f \left( {x}_{i} \right) \right] }{N} \times 100\%\end{eqnarray*}

(14) \begin{eqnarray*}FTS= \frac{\sum _{i}^{N}\cos \nolimits \left[ f \left( {x}_{i}+G \left( {x}_{i},{y}_{i} \right) \right) ,f \left( {y}_{i} \right) \right] }{N} \times 100\%.\end{eqnarray*}

### Experimental setup

**Dataset:** Our training set employs CASIA-WebFace (Yi et al., 2014), comprising 455,594 facial images of 10,575 distinct individuals after removing low-quality samples (blurred, occluded, or misaligned faces). We allocated 10% of the remaining data (stratified by identity) as the validation set. Our test set employs LFW (Huang et al., 2008), containing 13,233 images from 5,749 unique identities.

All samples across training, validation, and test sets underwent standardized preprocessing: (1) Face detection and alignment using MTCNN with five keypoints (eyes, nose, mouth corners), followed by (2) resizing to 112 ×112 pixels through bicubic interpolation to maintain consistency with input specifications of mainstream open-source face recognition models (*e.g.*, FaceNet, ArcFace).

**Model information:** In the training and testing process of AdvFaceGAN, multiple pre-trained FRModels by the GitHub project face-robustness-benchmark, and three commercial face recognition API models are used. The basic information of these models, the recognition threshold at 0.1%FAR, and the actual TAR and FAR calculated by using the training set CASIA-WebFace in this article to compare 6,000 random faces of homogeneity and non-homogeneity at each model recognition threshold can be described in Table 2.

**Table 2:** Face recognition model information.

Models	Basic structure	Loss function	Parameter count (M)	Recognition threshold	TAR/1-FAR
ArcFace	IR-SE50	ArcFace	43.8	0.284	98.9%/99.8%
FaceNet-VGGFace2	IncResV1	Triplet	27.91	0.421	98.9%/99.0%
MobileNet-stride1	MobileNet	CosFace	3.78	0.158	98.8%/99.4%
ShuffleNetV1	ShuffleNetV1	CosFace	1.46	0.191	99.0%/99.5%
ResNet50	ResNet50	CosFace	40.29	0.191	99.4%/99.8%
IR50-CosFace	IResNet50	CosFace	43.57	0.224	99.4%/99.9%
IR50-ArcFace	IResNet50	ArcFace	43.57	0.277	99.4%/99.9%
IR50-SphereFace	IResNet50	SphereFace	43.57	0.362	99.4%/99.6%
SphereFace	Shere20	SphereFace	28.08	0.349	97.5%/98.1%
CosFace	Shere20	CosFace	22.67	0.246	98.3%/98.3%
MobileFace	MobileFaceNet	CosFace	1.20	0.211	99.1%/99.6%
Face++ API				0.62327	99.3%/100%
Aliyun API				0.61	99.2%/100%
Tencent API				0.40	99.4%/100%

**Baseline methods:** This experiment compares three traditional gradient or optimization-based baseline methods FGSM (Goodfellow, Shlens & Szegedy, 2014), MI- FGSM (Kurakin, Goodfellow & Bengio, 2018) and C&W (Carlini & Wagner, 2017); locally perturbed baseline methods: Adv-MakeUP (Yin et al., 2021) and AT3D (Yang et al., 2023); globally perturbed baseline methods: AdvFace (Deb, Zhang & Jain, 2020), TIP-IM (Yang et al., 2023), SiblingAttack (Li et al., 2023) and DiffAM (Sun et al., 2024).

The principles followed when reproducing these comparison methods are: use the parameters from the original paper’s comparison experiments, or adjust the comparison methods to generate adversarial samples with a *L*_2_ norm perturbation that is close to the perturbation of the adversarial samples generated by the AdvFaceGAN model, which is 5. The specific model settings are as follows:

• **FGSM, MI-FGSM**: Use the implementation from the GitHub project ShawnXYang/Face-Robustness-Benchmark. The distance metric is set to the *L*_2_ norm, and the perturbation control parameter is set *ɛ* = 6, with other parameters as default. After verification, the adversarial samples generated with *ɛ* = 6 have a *L*_2_ norm perturbation close to 4.6, similar to the samples generated by our AdvFaceGAN model.
• **C&W**: Use the implementation from the GitHub project ShawnXYang/Face-Robustness-Benchmark. The perturbation control parameter is set *ɛ* = 16, with other parameters as default, and the code is modified to adjust the optimization objective to achieve 1.5 times the recognition threshold on the white-box surrogate model’s FTS, thereby enhancing the black-box attack capability.
• **AdvMakeUP**: Use the implementation provided in the paper author’s GitHub project Tencent/TFace. All parameters remain unchanged, *i.e.,* using the parameters from the original article for comparison experiments. However, the ensemble model used in the original article (irse50, facenet, and mobile_face) is modified to the models used by AdvFaceGAN (ArcFace, FaceNet, and ResNet50), and the dataset is changed from the original makeup dataset to the LFW dataset (using Face++’s face detection API to create the dataset keypoint files required by the code).
• **AT3D**: Use the implementation from the author’s GitHub open-source project thu-ml/AT3D. Refer to the AT3D-P parameter settings from the original paper’s digital-domain comparison experiment. The mask type is set to glasses + nose, with 300 iterations, and the perturbation control parameter is set *η* = 5 slightly higher than 3 (for stronger attack power). The perturbation generation algorithm is BIM.
• **AdvFaces**: Use the implementation from the paper author’s GitHub project ronny3050/AdvFaces. Using the parameters from the original paper for comparison experiments, with the perturbation control parameter *ɛ* = 8 as stated. However, the original FaceNet model is replaced with the FaceNet model used by AdvFaceGAN.
• **TIP-IM**: Use the implementation from the paper author’s GitHub project ShawnXYang/TIP-IM. All parameters remain unchanged, and AdvFaceGAN’s ArcFace, FaceNet, and ResNet50 models are used as surrogate models to generate adversarial samples.
• **SiblingAttack**: Use the implementation from the paper author’s GitHub open-source project Tencent/TFace. All parameters remain unchanged, *i.e.,* using the parameters from the original article for comparison experiments. After verification, the adversarial samples generated with these parameters have a *L*_2_ norm perturbation close to 10.9.
• **DiffAM**: Use the implementation from the author’s GitHub project HansSunY/DiffAM. The ensemble model used is changed from the original article’s irse50, facenet, and mobile_face to AdvFaceGAN’s ArcFace, FaceNet, and ResNet50, and the adversarial samples are generated using parameters that match the attack power mentioned by the author in the issues section.

**Experimental details:** In this experiment, the size of both the input source face and the output adversarial face is 3 × 112 ×112, and the batch size is set to 8; the Adam optimizer is used for training with parameters *β*_1_ of 0.5, *β*_2_ of 0.999, and the learning rate is set to 1e−4; the hyperparameter *λ*_*pert*_ = 1, *λ*_*adv*_ = 10 and *λ*_*st*_ = 20, which will make the various loss values equalize in magnitude, and is conducive to the effective convergence of the trained model on each loss.

### Loss ablation and parametric experiments

This section analyzes the significance of the perturbation loss *L*_*pert*_, structural loss *L*_*st*_, and source identity loss *L*_*adv*_*source*_ involved in the methodology of this paper, and constructs ablation experiments to analyze the specific effects they play. We also propose the specific numerical determination methods for the hyperparameters related to each loss: the upper limit of perturbation *ɛ*, the lower limit of structural similarity *ζ*, and the weight of source identity loss *η*.

**Ablation of** ***L***_***pert***_ **and determination of** *ɛ*

First, we tried *λ*_*pert*_ = *λ*_*st*_ = *μ* = 0. After training for 500 epochs, the model converged, but the Generator *G* chose to directly replace the source face with the target face. At this point, both the SSIM and MSE metrics were poor, and the FSS was very low, deviating from the premise that adversarial samples should maintain high similarity with the source face.

**Figure 6:**
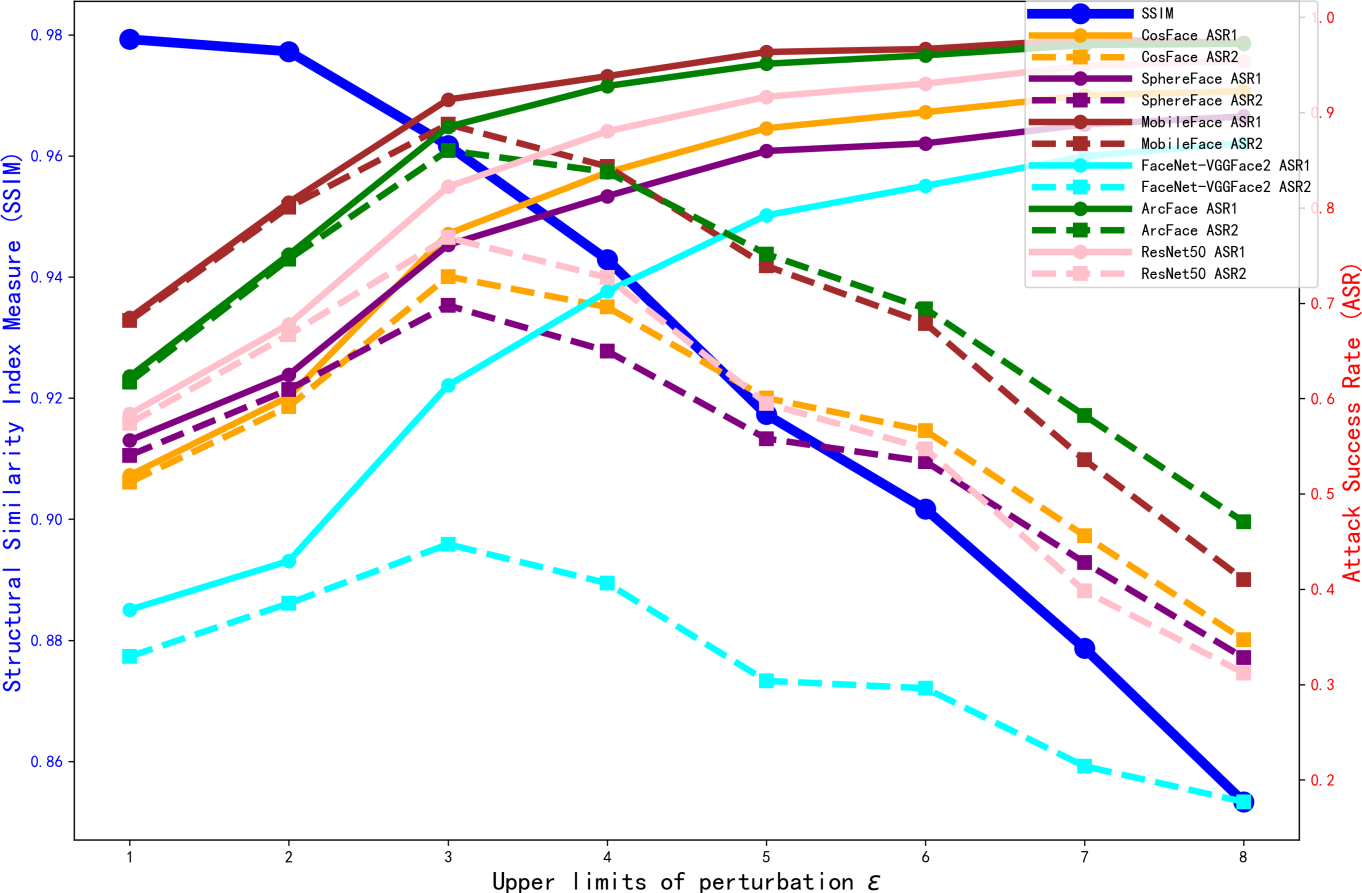
Comparison of ASR_1_ ASR_2_ and SSIM with different perturbation upper limit *ɛ*.

We sought to determine the value at which the perturbation upper limit *ɛ* should be set. Without adding the structure loss and source identity loss, we trained the model for 990 epochs under different perturbation upper limit *ɛ*. The ASR results for various offline face recognition models, as well as the SSIM metrics of the generated adversarial faces, are shown in Fig. 6.

**Figure 7:**
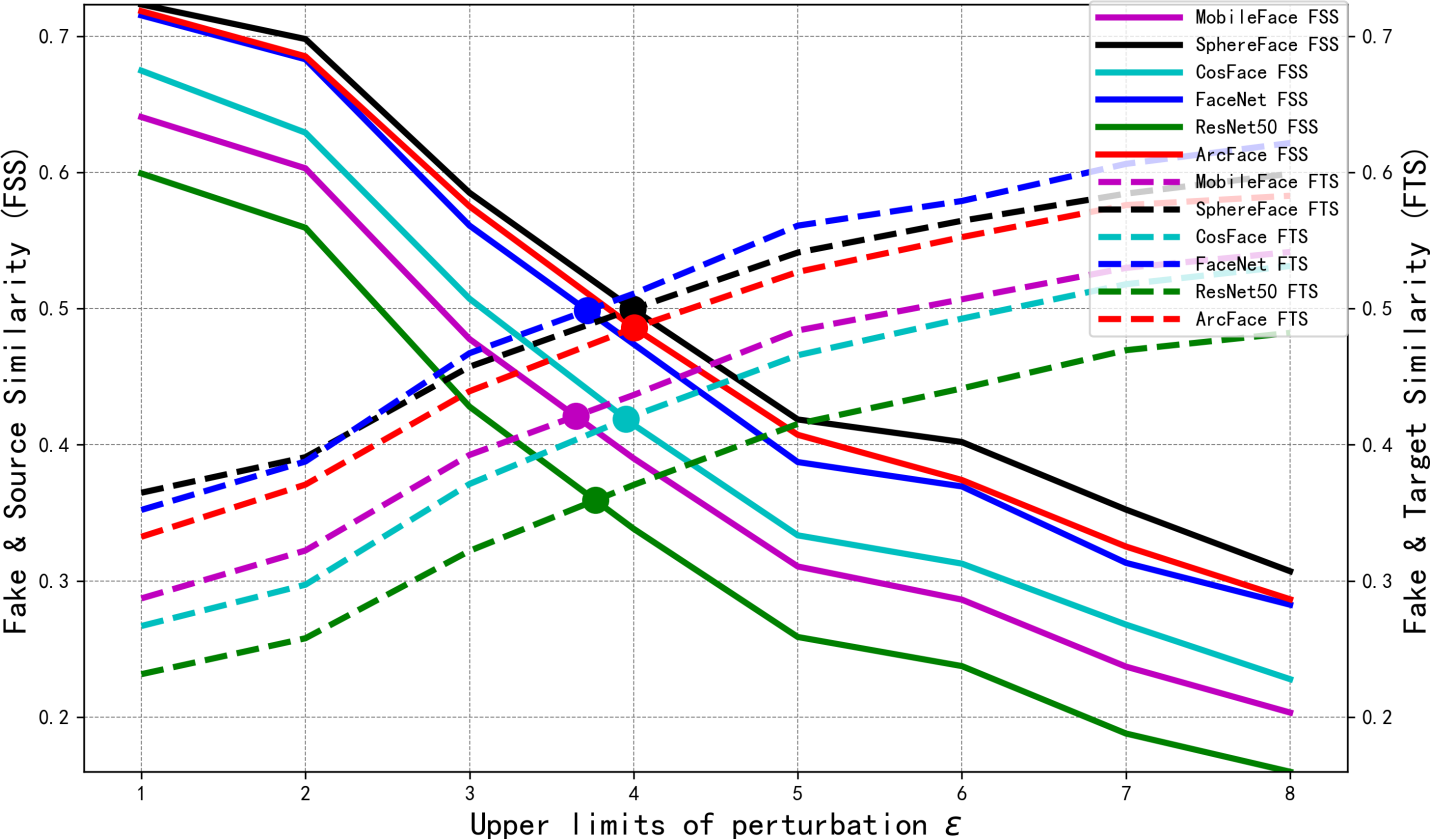
Comparison of FSS and FTS with different perturbation lower bounds *ɛ*.

As shown in Fig. 6, with the perturbation upper limit *ɛ* increases, the SSIM gradually decreases. Meanwhile, the values of each target model’s *ASR*_1_ increase and converge when the perturbation upper limit *ɛ* = 5. However, *ASR*_2_ shows an initial increase followed by a decrease. To investigate the reason for the decline in *ASR*_2_, we trained the model for 990 epochs under different perturbation upper limit *ɛ*. The FSS and FTS data of the 6,000 adversarial faces generated by each model in various face recognition models are shown in Fig. 7.

From Fig. 7, we can see that with the increase of the perturbation upper limit *ɛ*, the FTS gradually rises and the FSS gradually falls. At the same time, the FTS and FSS of various face recognition models roughly intersect at the perturbation upper limit *ɛ* = 4, when the generated antagonistic face has the optimal “dual identity” effect, and as shown in Fig. 6, the visual metric SSIM at *ɛ* = 4 is also relatively high, at 94%.

**Figure 8:**
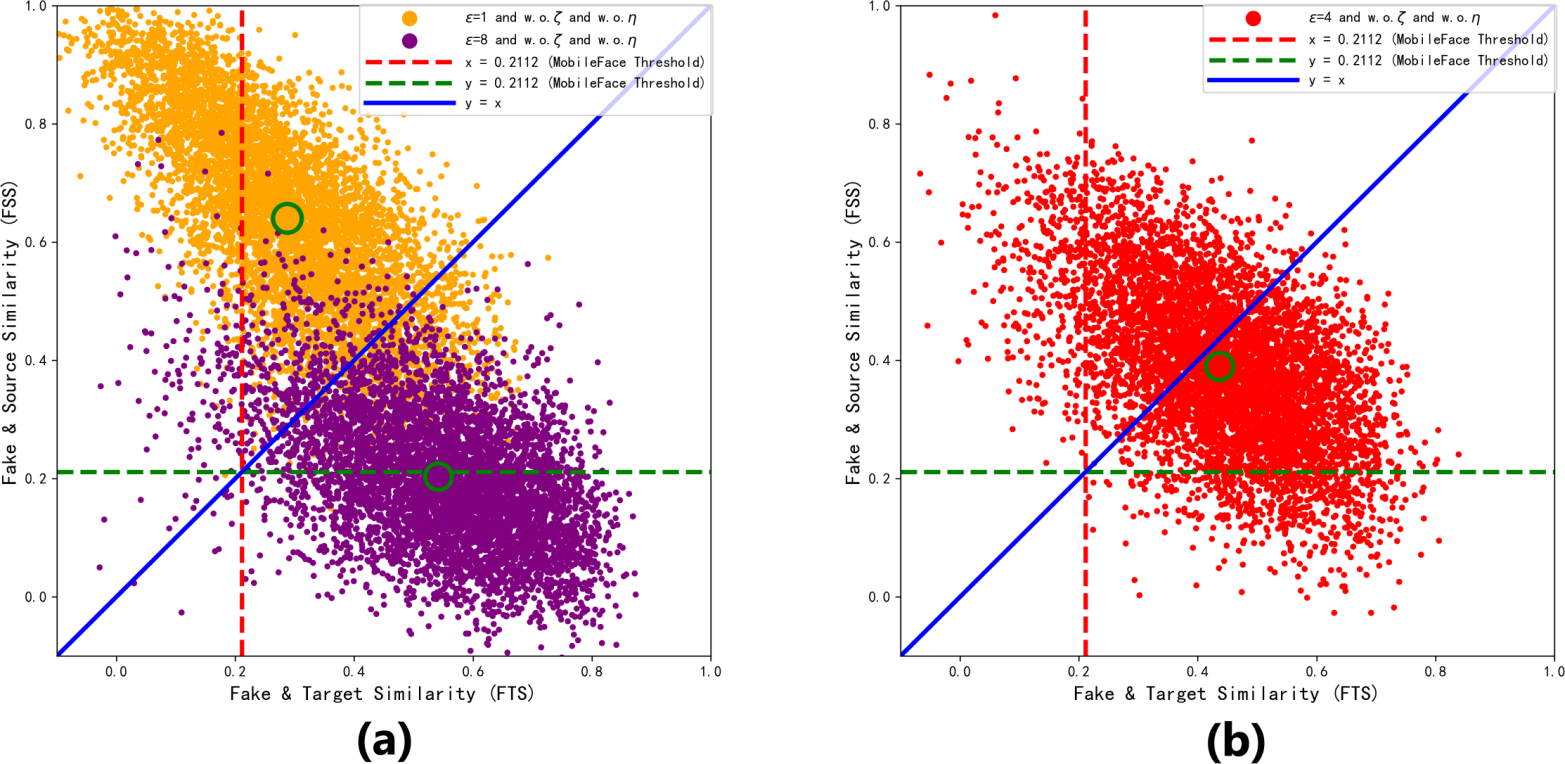
Distribution of the generated adversarial faces in the feature coordinate system with different perturbation upper limits *ɛ*. (A) *ɛ* =1 and *ɛ* =8 (B) *ɛ* =4.

In order to further visualize the superiority at *ɛ* = 4. We collect the FSS and FTS data of MobileFace from 6,000 adversarial faces generated under different perturbation upper limits *ɛ* trains 990 epoch, as shown in Fig. 8.

As shown in Fig. 8A, when *ɛ* = 1, the adversarial face is more likely to resemble the source identity, and when *ɛ* = 8, the adversarial face is more likely to resemble the target identity. From Fig. 8B, we can see that most of the adversarial faces generated when the perturbation amount is 4 are able to realize the attack effect above the threshold of “dual identity”, and realize the attack effect of resembling both the source identity and the target identity, so *ɛ* = 4 is the closest to the optimal solution at this time.

However, we find that adding the structure loss *L*_*st*_ or the source identity loss *L*_*adv*_*source*_ will cause the FSS to rise and the FTS to fall, and the equilibrium will be destroyed, and the experimental data when the target model is FaceNet-VGGFace2 and trains 990 epoch is shown in Table 3.

**Table 3:** Effect of adding structural loss or source identity loss on FSS *vs.* FTS.

Model info	SSIM	PSNR	MSE	*ASR*_1_	*ASR*_2_	FSS & FTS
*ɛ* = 5 *w*.*o*.*ζ* *w*.*o*.*η*	91.73	31.32	43.30	79.22	30.38	38.73 & 56.09
*ɛ* = 5 *ζ* = 0.92 *w*.*o*.*η*	94.50	32.38	34.92	75.48	39.33	45.57 & 53.09
*ɛ* = 5 *w*.*o*.*ζ* *η* = 0.15	92.72	31.46	42.16	75.48	42.25	47.82 & 53.36
*ɛ* = 5 *ζ* = 0.92 *η* = 0.15	94.62	32.57	33.72	70.57	43.92	51.76 & 50.96

**Figure 9:**
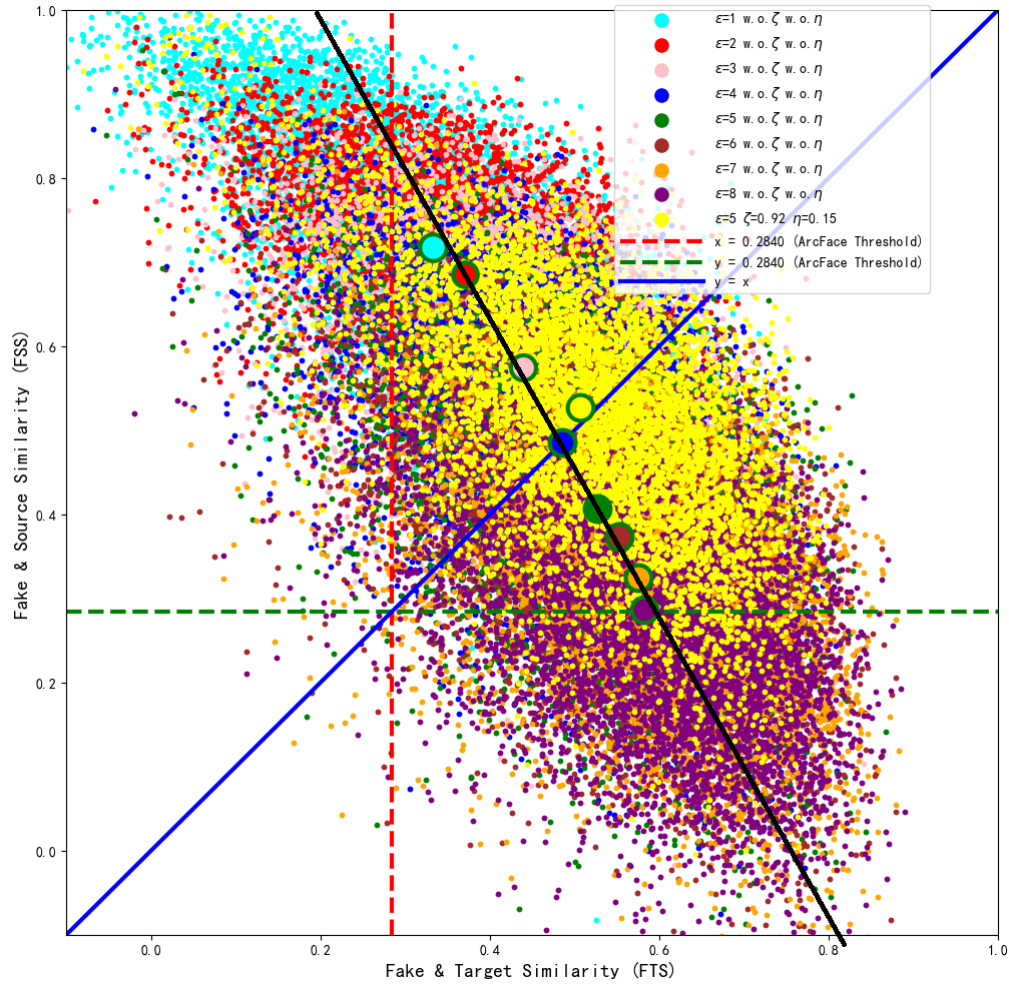
Comparison between the optimized model with perturbation lower bound *ɛ*=5 and the baseline model with *ɛ*=1–8.

Therefore, the perturbation upper limit *ɛ* needs to be set to the value in Fig. 7 where FTS is greater than FSS, *i.e., ɛ* ∈ (4, 8]. When the perturbation upper limit *ɛ* = 5, by adjusting *ζ* = 0.92 *μ* = 0.15, the model can converge and balance FSS and FTS again, which 51.76&50.96. As shown in Table 3, it can be seen that both the visual quality and *ASR*_2_ are the best. The optimized model *ɛ* = 5 *ζ* = 0.92 *η* = 0.15, with pixel points marked in yellow, was trained for 3,440 epochs, and after convergence, the generated samples were compared with the baseline model samples under different perturbation upper limits in the feature coordinate system of the ArcFace target model, as shown in the intuitive comparison in Fig. 9. It can also be observed that the generated adversarial samples achieved the best dual-identity effect.

**Ablation of** ***L***_***adv***_***source***_ **and determination of** *η*

From the data in Table 3, we observe the following: For the model with *ɛ* = 5 w.o. *ζ* w.o. *η*, after adding the source identity loss with *η* = 0.15, the FTS decreased 2.73 But the FSS increased 9.09. For the model with *ɛ* = 5 *ζ* = 0.92 w.o. *η*, after adding the source identity loss with *η* = 0.15, the FTS decreased 2.13 But the FSS increased 6.19. The exchange ratio is close to 1:3.

Thus, the addition of the source identity loss *L*_*adv*_*source*_ allows us to sacrifice a small decrease in FTS for a large increase in FSS. This will leads to a small decrease in *ASR*_1_ but a large increase in *ASR*_2_, as well as a slight improvement in visual quality.

The tuning method for the source identity loss weight *η* involves gradually increasing *η* and observing the FSS and FTS at model convergence until they balance. As shown in the data for *ɛ* = 5 w.o *ζ η* = 0.15, the FSS and FTS are 47.82 and 53.36. Therefore, to achieve a balance between FSS and FTS for the *ɛ* = 5 w.o *ζ* model, *η* should be increased further from the current value of 0.15.

**Ablation of** ***L***_***st***_ **and determination of** *ζ*

We collected experimental data under different structural similarity upper limits *ζ* with *ɛ* = 5 when the target model is FaceNet-VGGFace2 and trains 990 epoch, the specific data is shown in Table 4.

**Table 4:** Impact of the structural similarity lower limit *ζ*.

Model info	Training costs		Visual quality			Attack performance		
	TruePert	Epoch	SSIM	PSNR	MSE	ASR1	ASR2	FSS&FTS
*ɛ* = 4 w.o. *ζ* w.o. *η*	4.00	990	94.32	33.32	27.55	71.23	40.63	47.39&51.09
*ɛ* = 4*ζ* = 0.93 w.o. *η*	3.82	990	95.46	33.70	25.20	68.38	43.32	50.70&49.78
*ɛ* = 4*ζ* = 0.97 w.o. *η*	3.49	990	96.33	34.65	21.13	64.12	42.15	53.86&47.57
*ɛ* = 5 w.o. *ζ* w.o. *η*	5.01	990	91.73	31.32	43.30	79.22	30.38	38.73&56.09
*ɛ* = 5*ζ* = 0.92 w.o. *η*	4.50	990	94.50	32.38	34.92	75.48	39.33	45.57&53.09
*ɛ* = 5*ζ* = 0.95 w.o. *η*	3.97	990	95.62	33.52	27.80	69.53	38.30	48.41&50.23
*ɛ* = 5w.o.*ζ* *η* = 0.15	4.95	990	92.72	31.46	42.16	75.48	42.25	47.82&53.36
*ɛ* = 5*ζ* = 0.92*η* = 0.15	4.41	990	94.62	32.57	33.72	70.57	43.92	51.76&50.96
*ɛ* = 5*ζ* = 0.95*η* = 0.15	4.31	2490	95.13	32.70	32.50	73.57	48.03	52.35&52.23

From the data in Table 4, we included the TruePert (the *L*_2_ norm of the perturbation) and the Epoch (number of training epochs). The analysis reveals that after incorporating *L*_*st*_, the difficulty of perturbation learning significantly increases, even with extended training epochs to 2,490, the TruePert cannot converge to *ɛ*. This eliminated portion corresponds to perturbation patterns that strongly affect image structure, resulting in significant improvements across all visual metrics. Regarding attack metrics, similar to the source identity loss, we observe decreased FTS and increased FSS. Notably, the ratio of FTS decline to FSS increase is only about 1:1, substantially lower than the source identity loss approximate 1:3 exchange ratio. Furthermore, when *η* becomes excessive, FSS may surpass FTS. Continuing to increase *η* or adding source identity loss would exacerbate this imbalance (FSS > FTS), leading to *ASR*_2_ continues to decrease.

Therefore, the tuning method for the structure similarity lower limit *ζ* when *ɛ* = *x* is to set it to a value *y* larger than the SSIM of the baseline model *ɛ* = *x*, in order to remove perturbation patterns that strongly damage the image structure and enhance image quality. However, this value *y* cannot be too large, as it still needs to ensure that the *ɛ* = *x ζ* = *y* satisfy FSS < FTS, thus leaving optimization space for the source identity loss, for a more valuable exchange ratio.

In summary, the logic for determining the hyperparameters *ɛ*, *ζ* and *η* is as follows:

1. First, determine the perturbation upper limit *ɛ* which is strongly related to attack performance. Set it as a value *x* which FSS is less than FTS without adding the structural loss and source identity loss, from Fig. 7, *x* ∈ (4, 8] .
2. Then, with *ɛ* = *x*, determine the structural similarity lower limit *ζ* which is strongly related to visual quality. Set it as a value *y* which larger than the SSIM of the baseline model *ɛ* = *x* and ensuring that FSS is also smaller than FTS.
3. Finally, with *ɛ* = *x ζ* = *y*, determine the source identity loss weight *η*. Gradually increase *η* until FSS and FTS are balanced at convergence, which gives the final value *z*.

At this point, the three hyperparameters are determined, and the model trained with *ɛ* = *x ζ* = *y η* = *z* can achieve a good balance between attack performance and visual quality. As shown in Table 4, the model with *ɛ* = 5 *ζ* = 0.92 *η* = 0.15 reaches a balanced FSS and FTS of 52.35 and 52.23, and the *ASR*_2_ is the highest among various parameter comparisons.

### Ablation of ***FRs***

To explain part of the source of the attack performance of the method AdvFaceGAN in this article, we conducted an ablation experiment on the ensemble model FRs using MobileFace as the black-box target model. The ablation results for the ensemble model FRs are shown in Table 5 (where the hyperparameters for each model are set as *ɛ* = 5 *ζ* = 0.92 *η* = 0.15). Model *M*_1_ only uses FaceNet-VGGFace2 as the ensemble model; Model *M*_2_ adds more loss function-related models IR50-CosFace, IR50-ArcFace, and IR50-SphereFace on top of *M*_1_; Model *M*_3_ adds two new structural models, Mobilenet and ShuffleNet, on top of *M*_2_; Model *M*_4_ is the final ensemble model in this article, which adds the new structural models ArcFace and ResNet50 on top of *M*_3_.

**Table 5:** Ablation experiment of the ensemble model FRs.

Model info	Training costs		Visual quality			Attack performance		
	TruePert	Epoch	SSIM	PSNR	MSE	ASR1	ASR2	FSS&FTS
*M*_1_	4.49	1,490	95.02	32.37	34.81	69.47	68.53	60.71&34.06
*M*_2_	4.55	1,490	94.49	32.23	36.12	91.42	87.50	52.83&47.77
*M*_3_	4.56	1,490	94.40	32.26	36.10	91.83	87.90	53.62&48.39
*M*_4_	4.05	1,490	95.36	33.37	28.76	89.12	86.63	58.00&46.83
*M*_4_	4.32	2,490	95.14	32.70	32.57	92.28	89.65	55.63&49.25

By comparing the ASR of *M*_2_ and *M*_1_ in Table 5, it can be observed that integrating face recognition models with different loss functions provides the greatest improvement in attack effectiveness. Additionally, integrating face recognition models with different model structures also consistently enhances the attack effectiveness, although the model requires more training epochs to converge to the same perturbation amount as the complexity of the ensemble model increases.

Here, it should be mentioned that further adding face recognition models with higher recognition performance, which have different model structures or loss functions from those already integrated in the ensemble model, such as GhostFace, may lead to better attack effects, although the model convergence speed may further decrease.

### Comparison experiments

#### Offline model attack effectiveness

To demonstrate the superiority of AdvFaceGAN in terms of traditional impersonation attack success rate metrics, we conducted comparison experiments against several representative works using an offline model. In these comparison experiments, we selected gradient-based methods such as FGSM and MI-FGSM, optimization-based methods like C&W, as well as local perturbation attack methods such as AT3D and Adv-MakeUP, and global perturbation attack methods like SiblingAttack and AdvFace.

For the sake of experimental fairness and rigor, we referred to the experimental setups in the SiblingAttack works. We selected 110 faces from different categories in the LFW dataset as the test set, with 10 of them serving as the source faces and the remaining 100 as target faces. We reproduced all the comparison methods and conducted experiments on the same test set. We collected *ASR*_1_ for each method under both white-box and black-box scenarios, which is detailed in Table 6.

**Table 6:** Comparison of traditional impersonation attack success rates under white-box and black-box settings.

Method	White box attack			Black box attack		
	ArcFace	FaceNet	ResNet50	MobileFace	SphereFace	CosFace
FGSM	99.8%	85.4%	96.1%	64.8%	44.3%	40.8%
MI-FGSM	100%	100%	100%	97.6%	86.0%	80.2%
C&W	100%	100%	100%	7.1%	9.1%	8.1%
AdvMakeUP	25.0%	41.9%	31.7%	20.1%	37.1%	28.0%
AT3D	62.5%	84.7%	94.2%	88.6%	41.7%	67.0%
AdvFaces	86.1%	89.9%	78.1%	95.6%	84.5%	73.9%
TIP-IM	100%	99.8%	100%	89.1%	82.4%	91.0%
SiblingAttack	100%	100%	100%	99.2%	83.2%	92.7%
DiffAM	58.1%	36.7%	48.8%	65.6%	52.4%	38.2%
*ɛ* = 4 *w*.*o*. *ζ* *w*.*o*. *η*	95.7%	93.4%	95.9%	99.2%	79.5%	92.3%
*ɛ* = 5 *w*.*o*. *ζ* *w*.*o*. *η*	97.7%	97.8%	97.9%	99.3%	87.2%	96.8%
*ɛ* = 5 *ζ* = 0.92 *η* = 0.15	96.6%	94.8%	96.6%	99.2%	83.5%	94.4%

From the data in Table 6, we can see that MI-FGSM, as an improvement of the FGSM method, has significantly enhanced the attack performance in both white-box and black-box scenarios. In the white-box scenario, MI-FGSM achieves 100% attack success on ArcFace, FaceNet, and ResNet50. However, in the black-box scenario, it shows slight transferability issues, achieving only an 80.2% attack success rate on the CosFace model. The optimization-based method C&W also achieves 100% attack success in the white-box scenario, but its performance in the black-box scenario is severely impacted, with the attack success rate dropping below 10%, demonstrating the significant transferability limitations of optimization-based methods.

The core of SiblingAttack is based on the Projected Gradient Descent (PGD) algorithm. This method also achieves 100% attack success in the white-box scenario. Thanks to the use of gradient information from the face attribute recognition model ir152_ar, SiblingAttack overcomes the limitations of traditional gradient-based methods in terms of attack effectiveness and transferability in the black-box scenario, ranking just behind our method. AT3D and Adv-MakeUP are local perturbation methods that focus more on used in the physical world. They perform poorly in the digital domain for both white-box and black-box attacks against offline models. AdvFaces, as a GAN-based method, has good transferability, but because AdvFaces is based on WGAN-Clip, it suffers from poor convergence during training, resulting in a white-box attack success rate of less than 90%, and its performance in black-box attacks is also suboptimal.

In contrast, our method, although not achieving the 100% attack success rate in the white-box scenario due to the ensemble model, still achieves more than 95% attack success in the white-box scenario with a perturbation size of 5, without adding structural loss or source loss. Additionally, in the black-box scenario, our method demonstrates the advantages of GAN-based approaches in terms of transferability. For example, with a perturbation size of 5 and without structural loss or source loss, our method achieves the best black-box attack effectiveness and transferability among the comparison methods.

After adding structural loss and source identity loss, both black-box and white-box attack performance show slight degradation. However, this slight sacrifice in terms of traditional attack success rate is justifiable. We will present the comparison of dual-identity impersonation attack success rates in Table 7 to demonstrate that the minor trade-off in traditional impersonation attack success rates is worthwhile. It should be noted that, except for our method, none of the other methods consider similarity with the source identity.

**Table 7:** Comparison of dual-identity impersonation attack success rates under white-box and black-box settings.

Method	White box attack			Black box attack		
	ArcFace	FaceNet	ResNet50	MobileFace	SphereFace	CosFace
FGSM	99.7%	70.6%	96.1%	64.8%	44.3%	40.8%
MI-FGSM	6.3%	1.9%	37.8%	96.6%	84.5%	78.9%
C&W	100%	98.4%	100%	7.1%	9.1%	8.1%
AdvMakeUP	25.0%	41.9%	31.7%	20.1%	37.1%	28.0%
AT3D	60.7%	20.8%	82.1%	85.9%	40.4%	66.9%
AdvFaces	83.5%	58.8%	66.6%	33.7%	25.5%	27.5%
TIP-IM	0%	0%	0%	83.1%	76.7%	87.4%
SiblingAttack	0.9%	2.0%	2.5%	75.4%	72.5%	83.1%
DiffAM	35.9%	11.7%	30.5%	57.2%	42.2%	24.3%
*ɛ* = 4 *w*.*o*. *ζ* *w*.*o*. *η*	95.2%	73.9%	84.0%	92.7%	78.4%	91.6%
*ɛ* = 5 *w*.*o*.*ζ* *w*.*o*. *η*	94.5%	55.8%	67.1%	79.0%	82.3%	92%
*ɛ* = 5 *ζ* = 0.92 *η* = 0.15	96.2%	76.2%	88.6%	96.0%	81.8%	93.9%

From the data in Table 7, we can see that after adding structural loss and source identity loss, our method significantly improves the dual-identity impersonation attack success rate. In the white-box scenario, the success rate increases by 20% on FaceNet, and in the black-box scenario, it increases by 17% on MobileFace. Compared to other methods, our approach achieves the best white-box attack performance and black-box transferability in terms of dual-identity impersonation attack success rate.

At the same time, we also observe that other methods show various “misalignments” in terms of dual-identity impersonation attack success rate. This is because other methods do not consider similarity with the source identity during their design, and therefore, when generating adversarial faces, they do not take into account the key features that should be preserved to maintain similarity with the source face. Some methods, such as MI-FGSM and SiblingAttack, severely disrupt these key features in the white-box scenario, leading to poor performance in white-box attacks. However, since they do not disrupt features that are crucial for black-box models, their performance in black-box attacks is less affected. On the other hand, methods like C&W and FGSM cause less disruption to these features, which results in relatively stable performance in both white-box and black-box scenarios.

The similar method AdvFace exhibits the same performance as our method without the source identity loss and structural loss, as it does not consider the dual-identity impersonation attack success rate. As a result, its performance decreases. Local perturbation methods like AT3D and Adv-MakeUP, which do not modify a wide range of facial features, have relatively limited impact on attack performance. In terms of the data, Adv-MakeUP is not affected at all, while AT3D only experiences a slight impact.

Overall, our method has advantages over the comparison methods both in terms of traditional attack success rate and dual-identity attack success rate. Our method achieves the best black-box attack performance in terms of traditional attack success rate. In the dual-identity impersonation attack success rate, our method performs well in both white-box and black-box scenarios. By considering similarity with the source identity, our method avoids the “collapse” phenomenon observed in both white-box and black-box scenarios, which is present in some other methods.

### Visual quality comparison experiment

**Figure 10:**
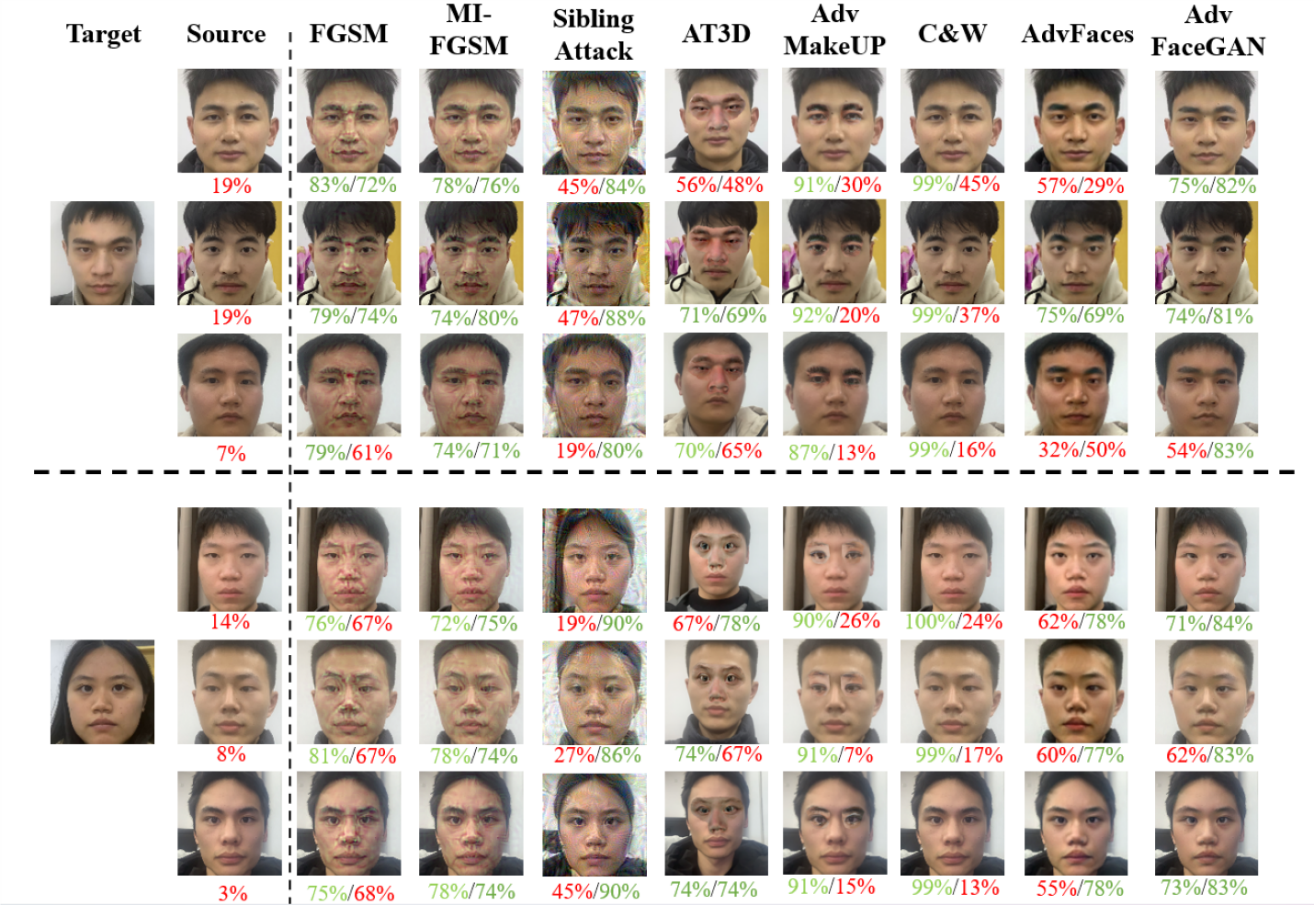
Visual comparison of adversarial samples generated by various attack methods.

To visually compare the quality of adversarial faces generated by each method, we selected five pairs of faces for a sample visual quality comparison experiment. The adversarial faces generated by each method are shown in Fig. 10. The two numbers below the face represent the feature similarity of the face to Source and Target, calculated by the Aliyun’s CompareFace API, with green representing above the 69% API threshold and red representing below the API threshold.

From Fig. 10, we can observe that gradient-based methods such as FGSM, MI-FGSM, and SiblingAttack, which is centered on the gradient method PGD, generate adversarial faces with poor quality. The perturbations are easily noticeable to the human eye. Local perturbation methods like AT3D and Adv-MakeUP, while modifying larger regions of the face, still generate noticeable adversarial patterns in local areas, which limits the visual quality of the adversarial faces and hinders their effectiveness in real-world attack scenarios. The C&W method generates adversarial faces with better visual quality; however, as we mentioned in previous experiments, C&W’s white-box attack performance is strong, but its black-box attack performance is nearly non-existent.

In contrast, adversarial faces generated by GAN-based methods such as AdvFace and AdvFaceGAN demonstrate better visual quality, highlighting the superiority of GAN-based approaches.

To quantitatively evaluate the visual quality of adversarial samples generated by different methods, we use ArcFace as the white-box substitute model to generate adversarial faces. We then calculate the attack success rate on the black-box model MobileFace. Additionally, we compute the SSIM, PSNR, and MSE metrics for the adversarial faces generated by each method to assess their visual quality quantitatively.

From the data in Table 8, it can be seen that our method, with *ɛ* = 5, *ζ* = 0.92 and *η* = 0.15, achieves better visual metrics than AT3D, SiblingAttack, Adv-MakeUP, and AdvFace. The SSIM of Adv-MakeUP is relatively high because it only generates makeup around the eyes in a small region, but its other two metrics are lower than ours, and its attack effectiveness is insufficient. Among traditional methods, the C&W method generates adversarial samples with unmatched visual quality, but its black-box attack transferability is very low, leading to a significant decrease in attack effectiveness. The MI-FGSM and FGSM methods slightly outperform our method in PSNR and MSE metrics, and MI-FGSM’s attack performance is also strong. However, when we use FaceNet as the white-box substitute model to generate adversarial faces and calculate the attack success rates in a black-box model (CosFace), as shown in Table 9, the transferability issues of FGSM and MI-FGSM are exposed. In contrast, our method maintains stable visual effects and attack performance, proving that it achieves the best visual quality among all the comparison methods under the same attack power and transferability conditions.

**Table 8:** Visual quality comparison of adversarial faces generated by different methods (ArcFace White-box, MobileFace Black-box).

Method	Vision metric			Attack metric		
	SSIM↑	PSNR↑	MSE↓	*ASR*_1_	*ASR*_2_	FSS&FTS
FGSM	90.2%	32.3	36.0	64.8%	64.8%	59.1&24.3
MI-FGSM	89.5%	32.2	36.3	97.6%	96.6%	43.6&39.7
C&W	99.5%	46.7	1.4	7.1%	7.1%	93.2&11.7
AdvMakeUP	97.4%	31.6	56.7	20.1%	20.1%	77.4&14.8
AT3D	89.6%	23.5	348.7	84.9%	82.5%	37.9&30.5
AdvFaces	91.6%	30.4	59.6	89.1%	83.1%	38.7&34.6
TIP-IM	85.3%	31.0	47.8	95.6%	21.5%	13.3&35.0
SiblingAttack	59.5%	24.9	205.2	99.1%	71.3%	27.7&50.5
DiffAM	82.4%	16.9	1367.0	65.6%	57.2%	32.3&24.9
*ɛ* = 4 *w*.*o*. *ζ* *w*.*o*. *η*	93.8%	33.3	28.1	99.2%	92.7%	38.3&49.6
*ɛ* = 5 *w*.*o*. *ζ* *w*.*o*. *η*	91.0%	31.3	43.9	99.3%	79.0%	30.2&54.3
*ɛ* = 5 *ζ* = 0.92 *η* = 0.15	94.0%	32.0	37.4	99.2%	96.0%	40.9&52.1

**Table 9:** Comparison of visual quality of adversarial faces generated by different methods (FaceNet White-box, CosFace Black-box).

Method	Vision metric			Attack metric		
	SSIM↑	PSNR↑	MSE↓	*ASR*_1_	*ASR*_2_	FSS&FTS
FGSM	88.4%	32.4	35.9	29.3%	29.3%	64.6&18.0
MI-FGSM	87.0%	32.3	36.3	69.5%	69.2%	53.5&31.2
C&W	99.4%	47.3	1.2	4.2%	4.2%	97.0&8.3
AdvMakeUP	97.4%	31.6	56.7	28.0%	28.0%	84.1&18.4
AT3D	88.4%	22.8	386.8	60.6%	60.2%	37.9&30.5
AdvFaces	91.6%	30.5	59.6	91.0%	87.4%	47.8&40.3
TIP-IM	83.7%	31.4	43.9	66.7%	27.5%	23.2&29.6
SiblingAttack	60.8%	25.1	196.4	92.7%	83.1%	40.3&42.5
DiffAM	82.4%	16.9	1367.0	38.2%	24.3%	31.5&22.3
*ɛ* = 4 *w*.*o*. *ζ* *w*.*o*. *η*	93.8%	33.3	28.1	92.3%	91.6%	54.8&40.6
*ɛ* = 5 *w*.*o*. *ζ* *w*.*o*. *η*	91.0%	31.3	43.9	96.8%	92.0%	46.1&46.4
*ɛ* = 5 *ζ* = 0.92 *η* = 0.15	94.0%	32.0	37.4	94.4%	93.9%	55.29&43.4

From Table 9, the insufficient transferability of FGSM and MI-FGSM attacks can be revealed, while the visual quality and attack efficacy of the proposed method remain stable. This demonstrates that our approach achieves the best visual quality among all compared methods under the same black-box impersonation attack efficacy.

### Face API attack effectiveness

This section will evaluate the black-box impersonation attack effectiveness on the face comparison APIs provided by Aliyun, Tencent, and Face++. Based on previous experience with black-box attacks on offline models using three white-box models, it was found that when using ArcFace as a white-box substitute model, various gradient-based methods achieved the best black-box attack success rate for offline models. Therefore, the adversarial samples generated using different methods with ArcFace as the white-box substitute model in the previous Table 6 (offline model black-box attack experiments) will be used to evaluate these commercial APIs.

Additionally, the three face comparison APIs each provide three recommended recognition thresholds. For example, Aliyun’s recommended thresholds are [61, 69, 75], Tencent’s recommended thresholds are [40, 50, 60], and Face++’s recommended thresholds are [62.327, 69.101, 73.975]. These thresholds correspond to the recognition thresholds that achieve 0.1% FAR, 0.01% FAR, and 0.001% FAR for each commercial API. However, a higher false acceptance rate (FAR) also leads to a decrease in the true acceptance rate (TAR). Therefore, this section of the experiment first uses the recommended threshold at 0.01% FAR for all three APIs to determine whether the impersonation attack is successful. The data obtained is shown in Table 10.

By examining the data in Table 10, it can be observed that the proposed method, Our method performs slightly worse than SiblingAttack in terms of the traditional impersonation attack success rate *ASR*_1_, but the dual-identity impersonation attack success rate is higher than all comparison methods. Additionally, the attack effectiveness remains stable across the three commercial APIs, demonstrating that the adversarial samples generated by AdvFaceGAN have excellent transferability.

Other similar studies that evaluate the success rate of attacks on commercial APIs often use the 0.1% FAR threshold, which is less secure and more vulnerable to attacks. This article also provides additional attack data at the recommended threshold for 0.1% FAR for each method, as shown in Table 11.

**Table 10:** 0.01% FAR threshold: black-box attack success rate comparison on Aliyun, Tencent and Face++.

Metric	APIs	FGSM	MI-FGSM	C&W	Adv- MakeUP	AT3D	AdvFace	TIPIM	Sibling attack	DiffAM	Adv FaceGAN
*ASR*_1_	Aliyun	5.0%	36.7%	0%	0%	11.2%	21.6%	20.1%	82.0%	0%	79.3%
	Tencent	21.1%	51.2%	1.7%	4.9%	41.6%	41.1%	46.3%	85.2%	11.5%	76.5%
	Face++	27.4%	67.2%	2.7%	5.6%	59.0%	60.0%	57.2%	92.0%	14.1%	89.8%
*ASR*_2_	Aliyun	5.0%	34.3%	0%	0%	5.7%	11.9%	3.5%	16.5%	0%	62.1%
	Tencent	21.1%	50.1%	1.7%	4.9%	25.2%	29.3%	22.8%	39.5%	3.6%	58.4%
	Face++	27.4%	65.2%	2.7%	5.6%	30.2%	39.9%	15.3%	51.5%	2.2%	68.3%

**Table 11:** 0.1% FAR threshold: black-box attack success rate comparison on Aliyun, Tencent and Face++.

Metric	APIs	FGSM	MI-FGSM	C&W	Adv- MakeUP	AT3D	AdvFace	TIPIM	Sibling attack	DiffAM	Adv FaceGAN
*ASR*_1_	Aliyun	25.2%	72.3%	0.2%	1.2%	48.0%	45.6%	58.7%	94.7%	3.7%	93.5%
	Tencent	40.2%	67.3%	6.1%	15.8%	68.7%	59.2%	66.0%	92.7%	30.5%	88.2%
	Face++	46.9%	77.5%	9.2%	14.3%	79.9%	79.9%	73.8%	96.1%	31.5%	95.3%
*ASR*_2_	Aliyun	25.2%	71.9%	0.2%	1.2%	38.9%	38.0%	24.7%	47.5%	1.7%	89.6%
	Tencent	40.2%	67.0%	6.1%	15.8%	53.4%	50.6%	46.8%	60.5%	18.7%	80.3%
	Face++	46.9%	77.0%	9.2%	14.3%	55.1%	66.3%	38.6%	72.5%	11.8%	84.4%

By examining the attack data in Table 11 at the 0.1% FAR threshold, it can be observed that the conclusions remain consistent with those at the 0.01% FAR threshold. However, it is noteworthy that the decline in *ASR*_2_ compared to *ASR*_1_ is less pronounced for the other methods. This is because methods that do not consider the source identity loss tend to generate adversarial faces with higher FTS but lower FSS. At a lower recognition threshold *τ*, the negative impact of low FSS on *ASR*_2_ isreduced. This can be understood by referring to Eq. (12). In essence, the lower FSS weakens the detrimental effect on *ASR*_2_at lower thresholds, which helps explain the observed differences in attack performance at varying FAR levels.

Therefore, setting a higher recognition threshold not only enhances the resistance to traditional impersonation attacks but also improves the resistance of commercial APIs to dual-identity impersonation attacks.

### Dual-identity impersonation attack failure pattern diagnosis

**Figure 11:**
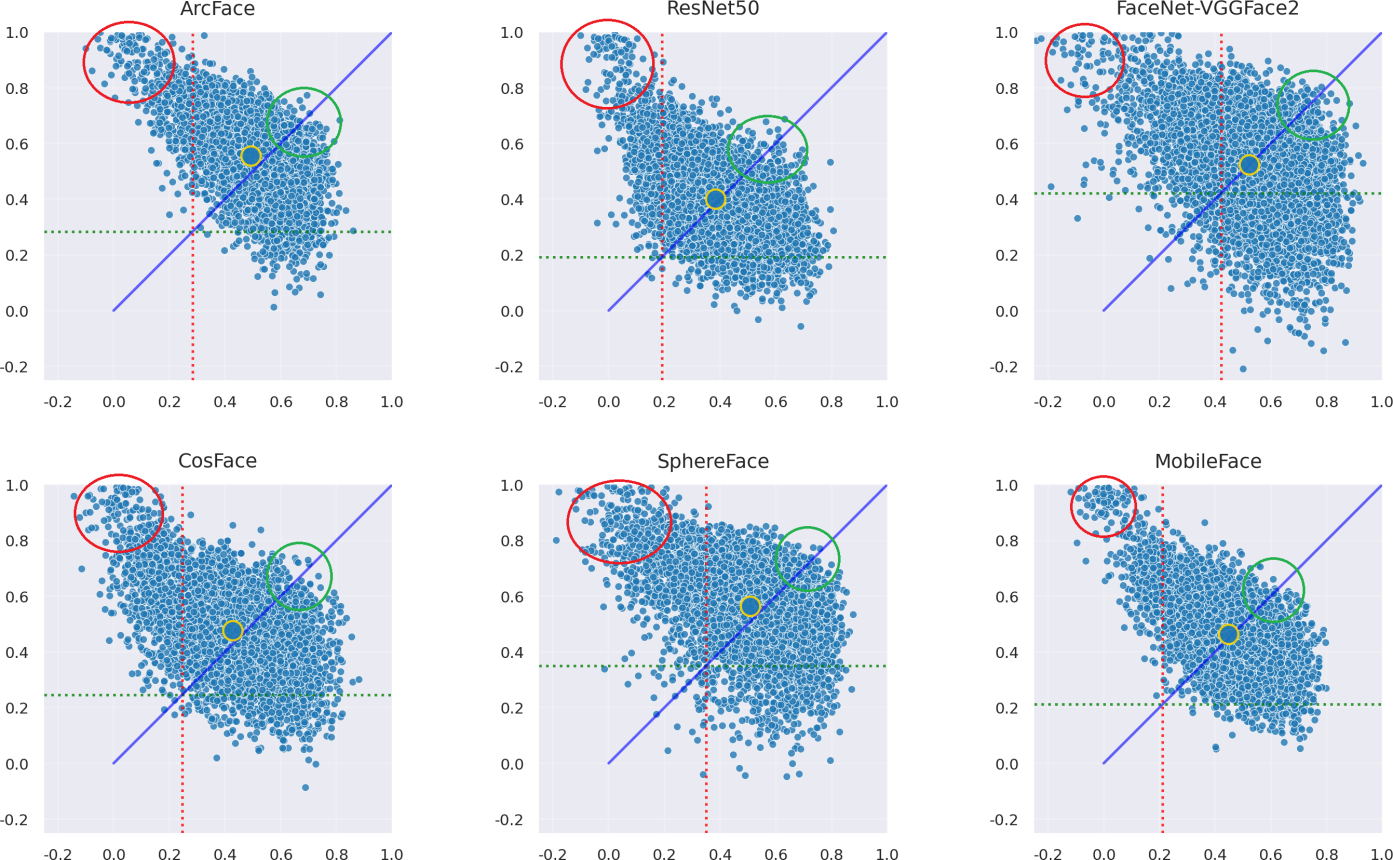
Distribution of AdvFaceGAN adversarial samples.

This section of the experiment analyzes whether there are certain situations in which the adversarial samples generated by the AdvFaceGAN method in this article lead to difficulties in successful attacks. First, the distribution of the adversarial samples generated by the AdvFaceGAN method in the feature coordinate systems of three white-box models and three black-box models is plotted, as shown in Fig. 11.

**Figure 12:**
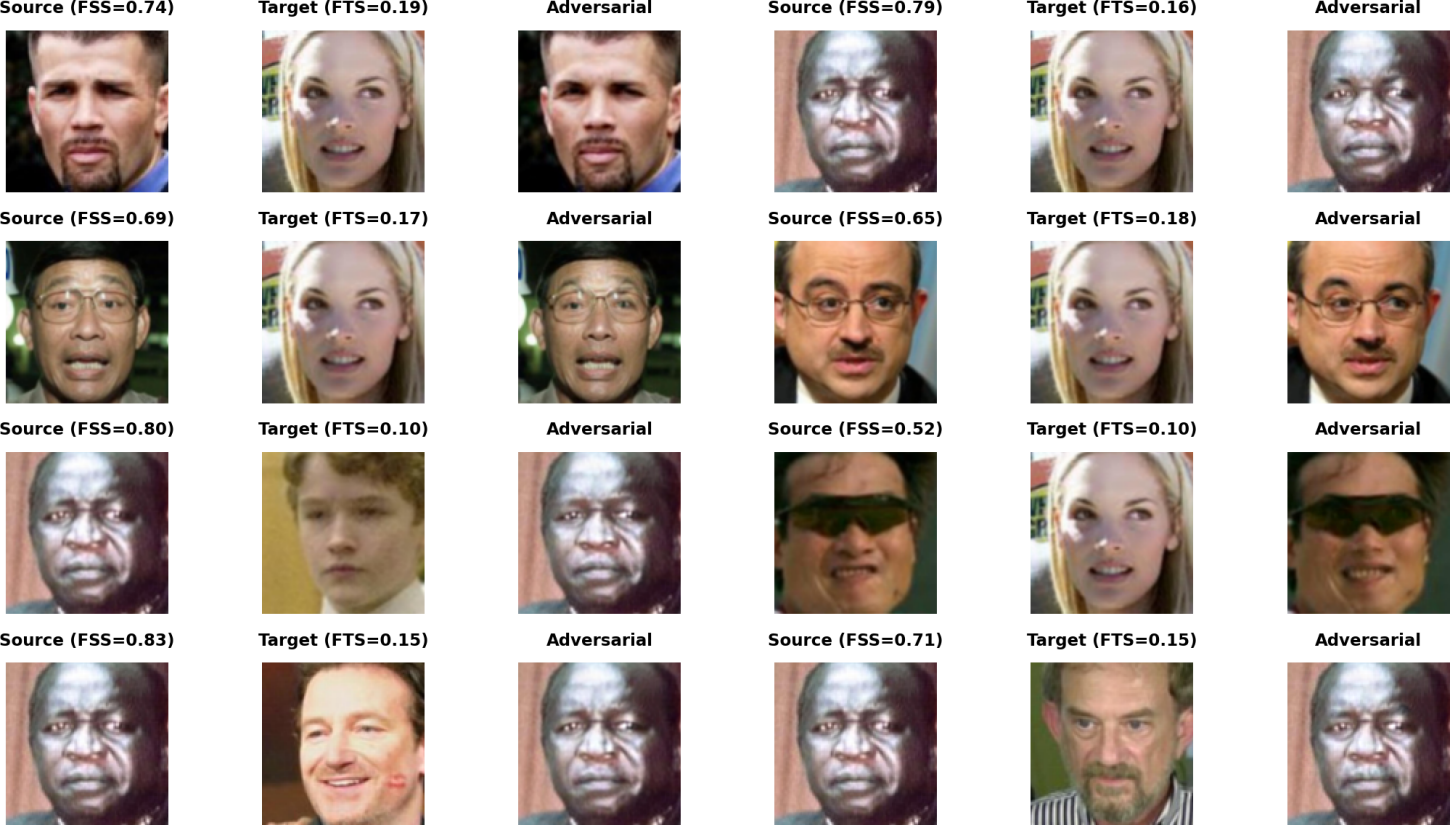
Visualization of failed AdvFaceGAN adversarial samples.

As shown in Fig. 11, some samples in the top-left corner are discarded during the adversarial network optimization process and fail to achieve the dual-identity effect. These samples are visualized when attacking MobileFace in Fig. 12.

As shown in Fig. 12, it can be observed that when the source face and target face involve cross-skin tone or cross-gender situations, FTS struggles to reach the correct value, thus failing to achieve the dual-identity impersonation attack.

**Figure 13:**
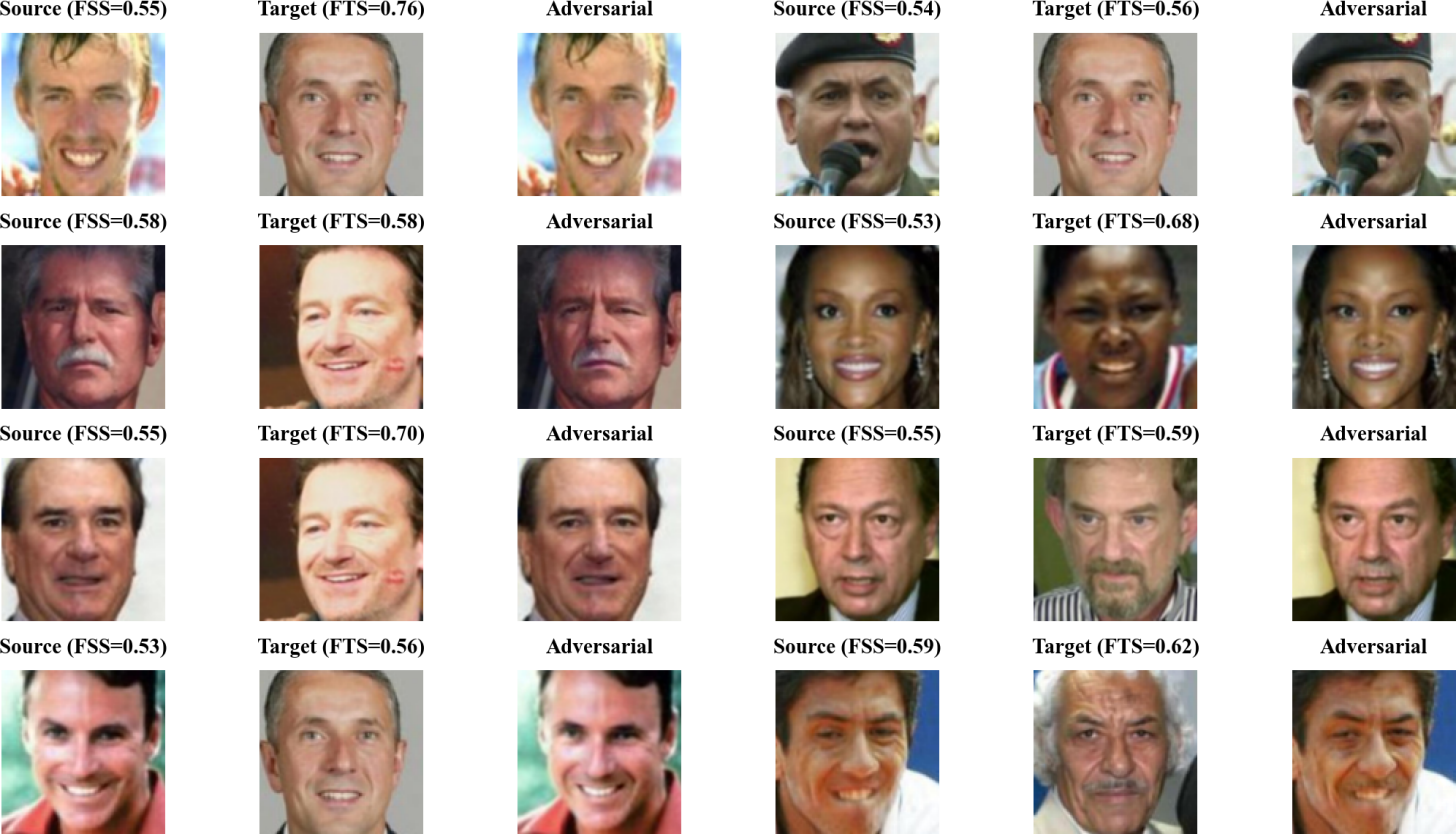
Visualization of excellent AdvFaceGAN adversarial samples.

As shown in Fig. 11, it can be observed that the samples within the green circle in the upper right achieved the highest FSS & FTS during the optimization process of the Generative Adversarial Network, demonstrating excellent dual identity effects. The visualization of these samples when attacking MobileFace is shown in Fig. 13.

This suggests that attackers need to select victims whose gender and skin tone are similar to their own in order to improve the success rate of dual-identity impersonation attacks.

### Time processing experiments

This section compares the time cost required by different attack methods for generating adversarial faces for a new source identity and target identity. The attack methods are divided into Algorithm-based and GAN-based approaches. Algorithm-based attack methods do not require pre-training of neural networks, so there is no training cost. For GAN-based attack methods, Adv-MakeUP and DiffAM need to re-train the network for a new target identity, so the total cost is the sum of training cost and inference cost. AdvFaces and the method presented in this paper, AdvFaceGAN, do not require re-training the network for a new target identity, so the total cost is the inference cost. The time is measured in seconds, and the timing for this experiment was conducted on a laptop with an i9-14900 CPU and an RTX4070 GPU configuration, Only DiffAM was trained on a cloud server with 24 GB of VRAM due to its maximum requirement of nearly 21.398 GB of VRAM.

As shown in Table 12, Algorithm-based attack methods such as C&W and SiblingAttack often require more time for multi-step iterations to achieve better attack results, making them much slower than AdvFaceGAN, which only requires inference. The training cost for GAN-based methods is faster than that of AdvFaceGAN because Adv-MakeUP and DiffAM train networks that can only generate adversarial faces for specific target identities, and AdvFaces uses FaceNet as the only one substitute model. These methods have simpler training objectives. However, AdvFaceGAN does not require re-training for new target identities, so when generating adversarial faces for new source and target identities, The total cost for AdvFaceGAN is only 0.25 s, which is fast enough.

**Table 12:** Comparison of time costs for different attack methods.

Type	Method	Training cost (s)	Inference cost (s)	Total cost (s)	
Algorithm-based	FGSM		0.11	0.11	
	MI-FGSM		0.16	0.16	
	C&W		14.71	14.71	
	TIP-IM		9.83	9.83	
	AT3D		8.95	8.95	
	SiblingAttack		114.23	114.23	
GAN-based	Adv-MakeUP	43,632	0.37	43,633.24	
	DiffAM	1,558	3.95	1,562.59	
	AdvFaces	41,508	0.12	0.12	
Our model	AdvFaceGAN	483,301	0.25	0.25	

## Discussion

This article, based on the potential security issues in real-world applications such as facial recognition attendance systems, demonstrates that generating “dual-identity” adversarial faces and uploading them to facial databases to bypass liveness detection mechanisms, achieving a high success rate in impersonation attacks, is both realistic and feasible. The proposed method, AdvFaceGAN, ensures the visual concealment of adversarial faces through multiple vision-related losses and enhances the attack and transferability capabilities by constructing an ensemble of facial white-box models with maximum model differences. The novel source identity loss introduced in this method ensures that, during the optimization process whered adversarial faces remain similar to the target face, sufficient similarity to the source identity is maintained. Experimental results show that AdvFaceGAN achieves higher visual concealment and transfer attack effectiveness compared to existing similar attack methods, and its performance in dual-identity impersonation attack scenarios far exceeds current state-of-the-art methods.

Experiments on both open-source offline models and commercial APIs indicate that facial recognition systems without defenses against adversarial samples are highly vulnerable. The AdvFaceGAN method still achieves around 60% attack success rate on three commercial APIs at a 0.01% FAR, demonstrating that current commercial APIs have yet to design effective defenses against adversarial attacks. Current defenses in facial recognition systems can focus on preventing direct attacks in the digital domain, such as preventing administrators from uploading facial images of employees to attendance systems. For example, systems could be designed to collect real-time facial images using a camera through a mini-program for employee registration.

In the design of AdvFaceGAN, we focus on finding more concealed perturbations to evade detection by the human eye. However, the attack currently only works in the digital domain, and its application in the physical world still depends on utilizing the dual-identity characteristics for uploading to facial databases. A further challenge to address is how to directly attack in the physical world. In the next step, this research could explore applying perturbations to a 3D mask using methods similar to those used in AT3D, adapting to the complexities of the physical world to directly achieve the attack effect.

## Additional Information and Declarations

### Data Availability

The following information was supplied regarding data availability: The original code is available at GitHub and Zenodo: - https://github.com/yangyang-onlysky/AdvFaceGAN
- Yang, . yang . (2025). AdvFaceGAN 1.0.1 for Peerj (1.0.1). Zenodo. https://doi.org/10.5281/zenodo.14922725
The data are available at Figshare: - yang, yang (2024). lfw-aligned-112x112. figshare. Dataset. https://doi.org/10.6084/m9.figshare.27073438.v1
- yang, yang (2024). casia-aligned-112x112. figshare. Dataset. https://doi.org/10.6084/m9.figshare.27073465.v1.
